# Supplementary material for: Intracranial injection of genetically modified, mosquito non-transmissible Zika virus: Safety in primates and ramifications for brain tumor therapy
Source: Cell Rep Med. 2025 Dec 16;6(12):102509. doi: 10.1016/j.xcrm.2025.102509 (PMC12765944; doi:10.1016/j.xcrm.2025.102509)
Supplement: Document S1. Figures S1–S4 [file mmc1.pdf]

Cell Reports Medicine, Volume *6*

Supplemental information

Intracranial injection of genetically modified, mosquito non-transmissible Zika virus: Safety in primates and ramifications for brain tumor therapy

Alec J. Hirsch, Amanda de Andrade Costa, Cody German, Christopher J. Parkins, Jessica L. Smith, Emilie Russler-Germain, Ashwani Kesarwani, Yuping Li, Verginia Cuzon Carlson, Timothy Carlson, Jodi L. McBride, Sathya Srinivasan, Anne D. Lewis, Xuping Xie, Pei-Yong Shi, Michael S. Diamond, and Milan G. Chheda

Supplementary Figures

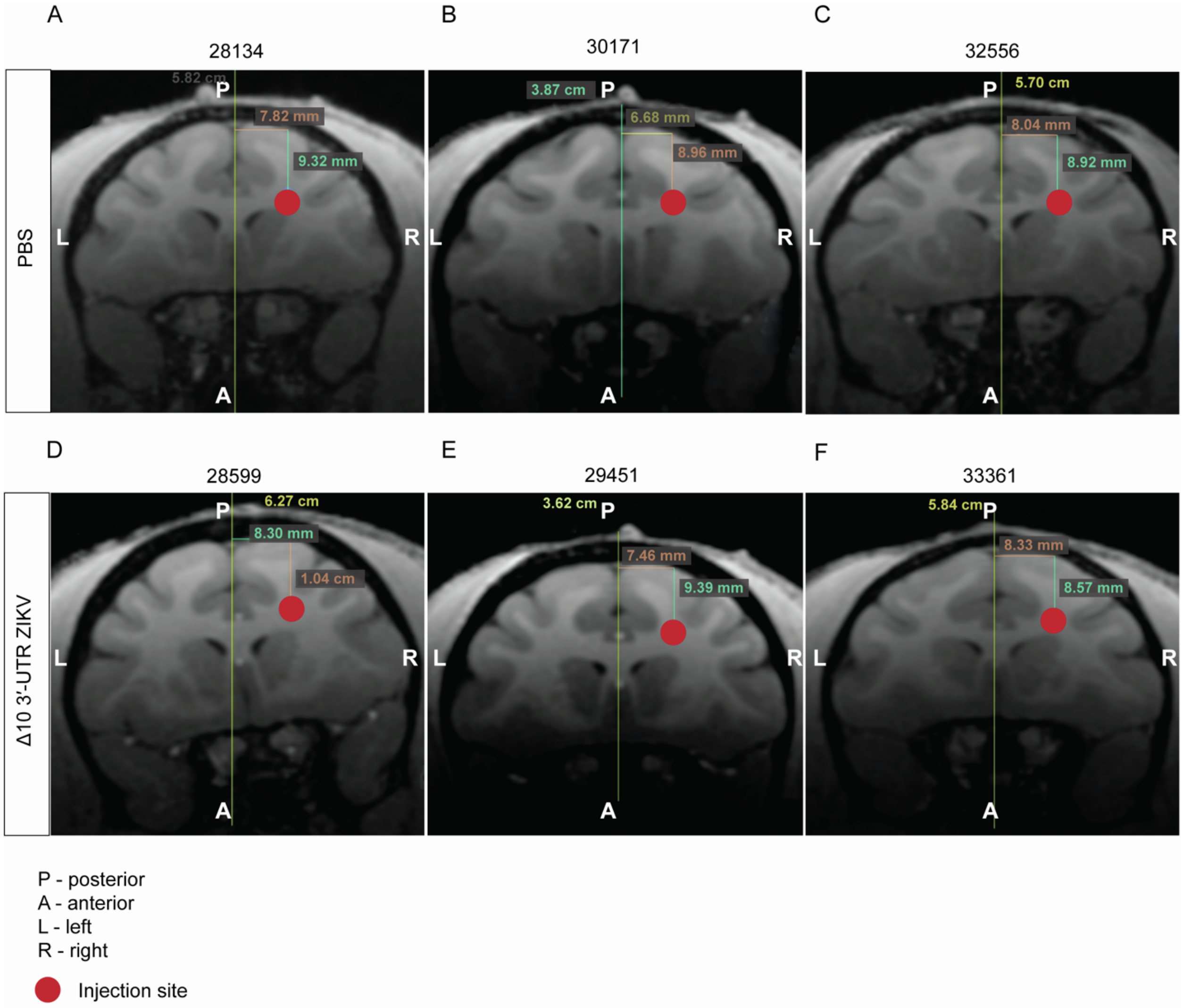

Figure S1. MRI-guided injection coordinates. MRI scan indicating the exact coordination for injection of PBS (A, B, C) or Δ10 3′-UTR ZIKV (D, E, F) for animals A. 28134 B. 30171 C. 32556 D. 28599 E. 29451 F. 33361. Related to Figure 1.

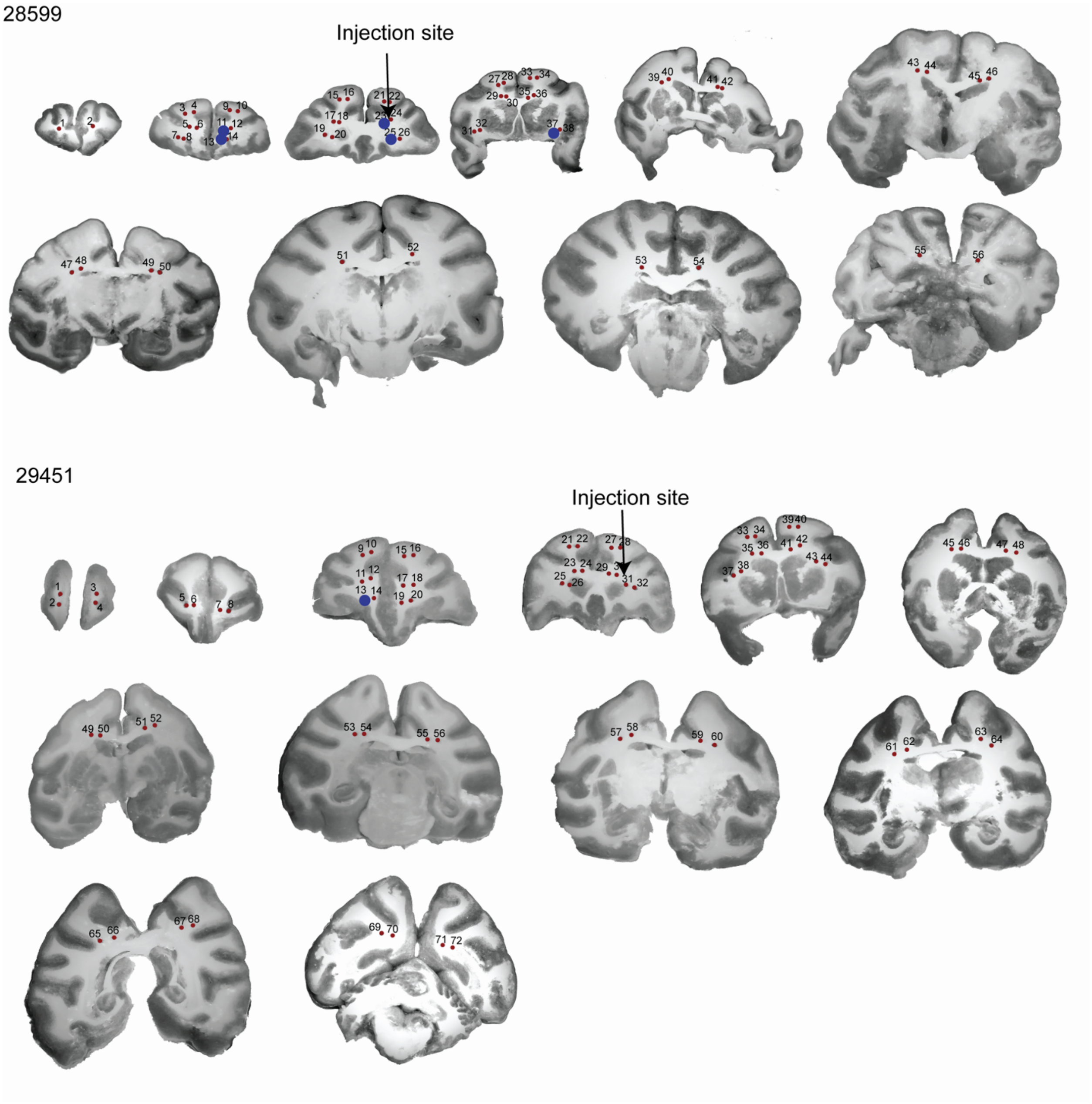

Figure S2. Representation of brain slabs from ZIKV injected macaques with numbered sampling sites indicated. Related to Fig 2.A.

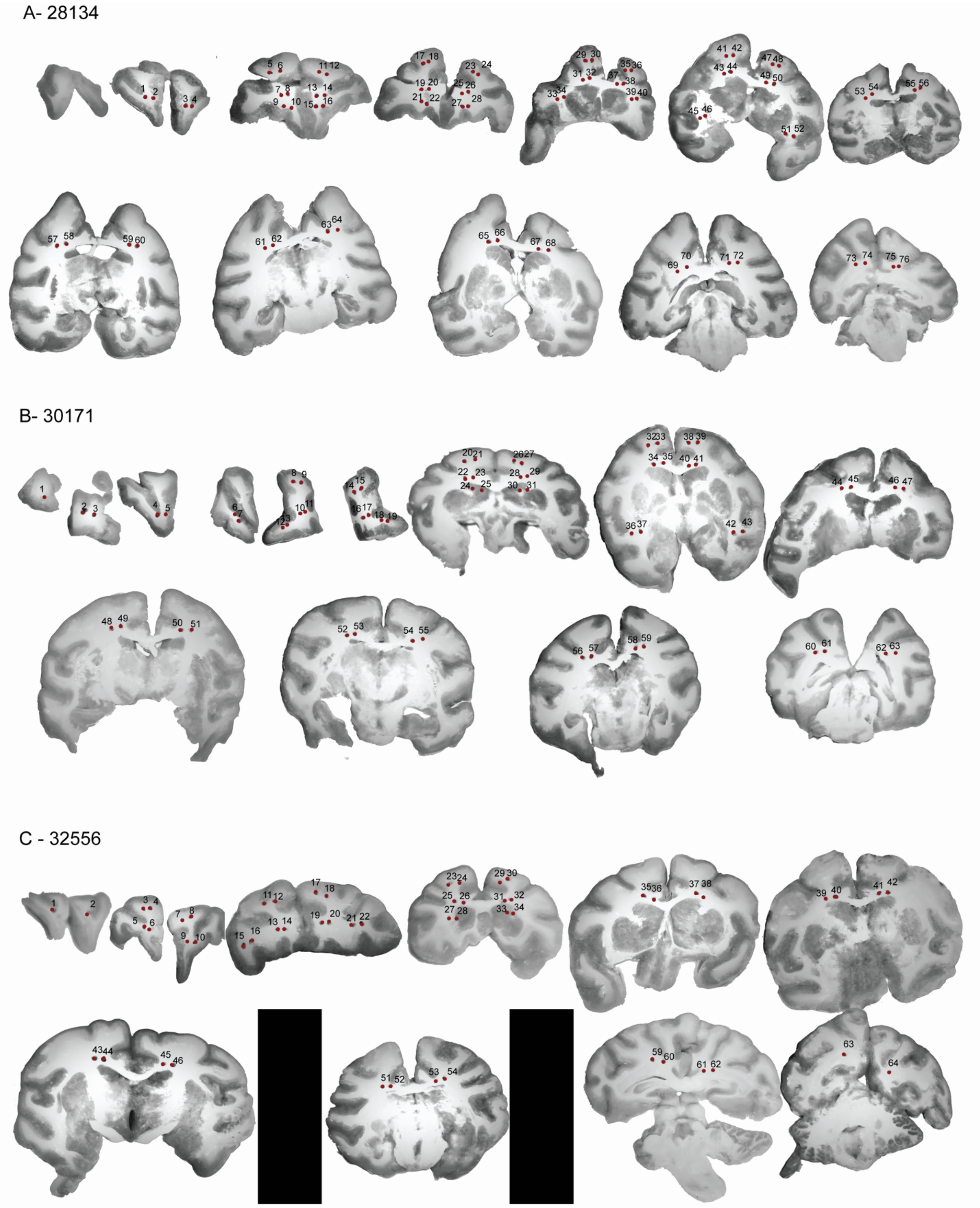

Figure S3. Representation of brain slabs from PBS injected macaques with numbered sampling sites indicated. Related to Figure 2A.

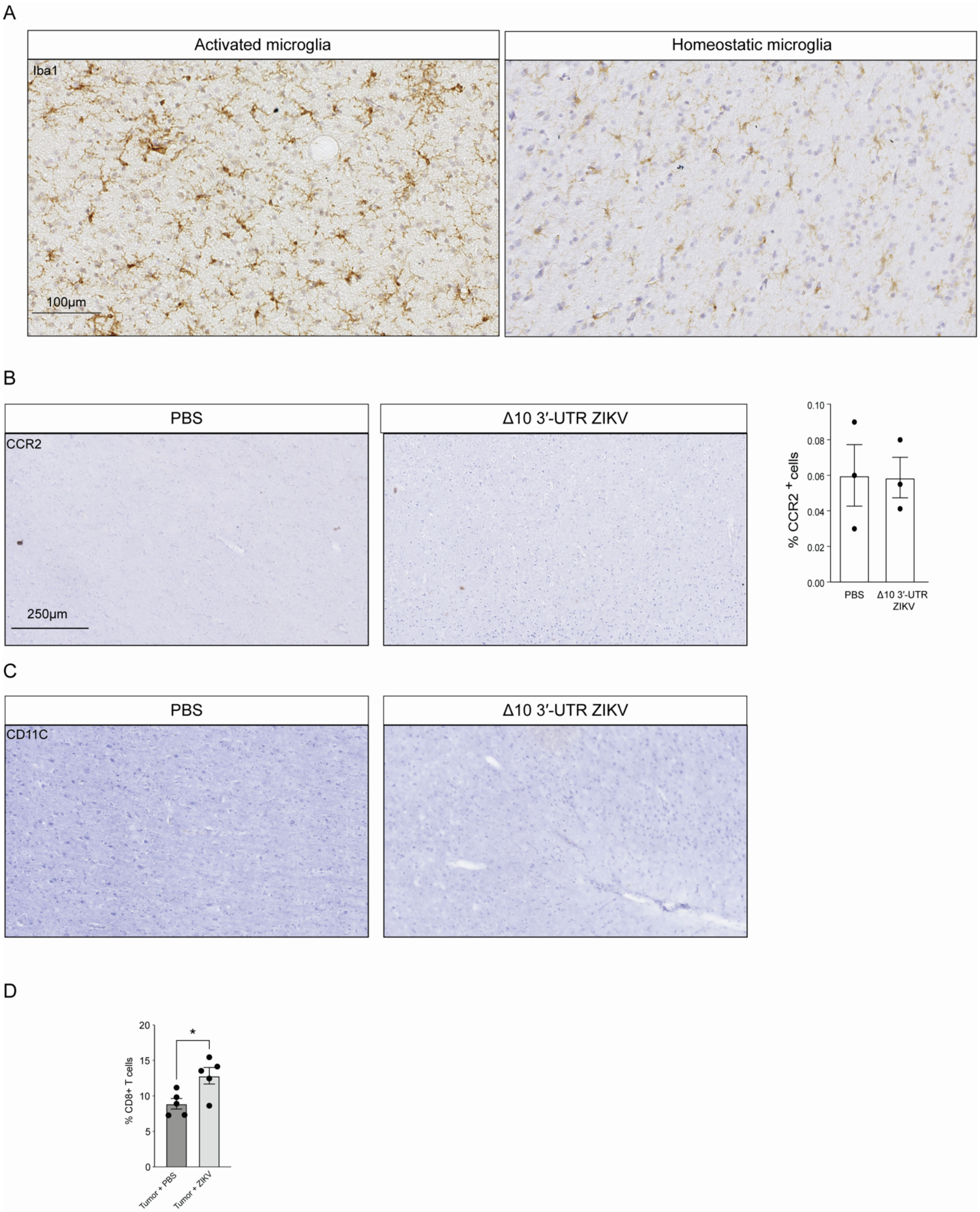

Figure S4. Immune cell populations in the brain after intracerebral ZIKV injection.

A. IBA1 immunostaining of Δ10 3′-UTR ZIKV injected macaques demonstrated focal areas where microglia showing an activated phenotype (left panel) versus their basal

morphology (right panel). **B.** Quantification of CCR2-expressing cells in the brains of NHP after intracranial injection of PBS or ZIKV. **C.** Quantification of CD11c-expressing cells in the brains of rhesus macaques after intracranial injection of PBS or ZIKV. **D.** Quantification of expression of CD8-expressing cells in the brains of na.ve mice, and brains with or without tumors 14 days after ZIKV injection. Related to Figure 3.
