# Supplementary material for: Intracranial injection of genetically modified, mosquito non-transmissible Zika virus: Safety in primates and ramifications for brain tumor therapy
Source: Cell Rep Med. 2025 Dec 16;6(12):102509. doi: 10.1016/j.xcrm.2025.102509 (PMC12765944; doi:10.1016/j.xcrm.2025.102509)
Supplement: Document S2. Article plus supplemental information [file mmc2.pdf]

Cell Reports
Medicine

Report

Intracranial injection of genetically modified, mosquito non-transmissible Zika virus: Safety in primates and ramifications for brain tumor therapy

Graphical abstract

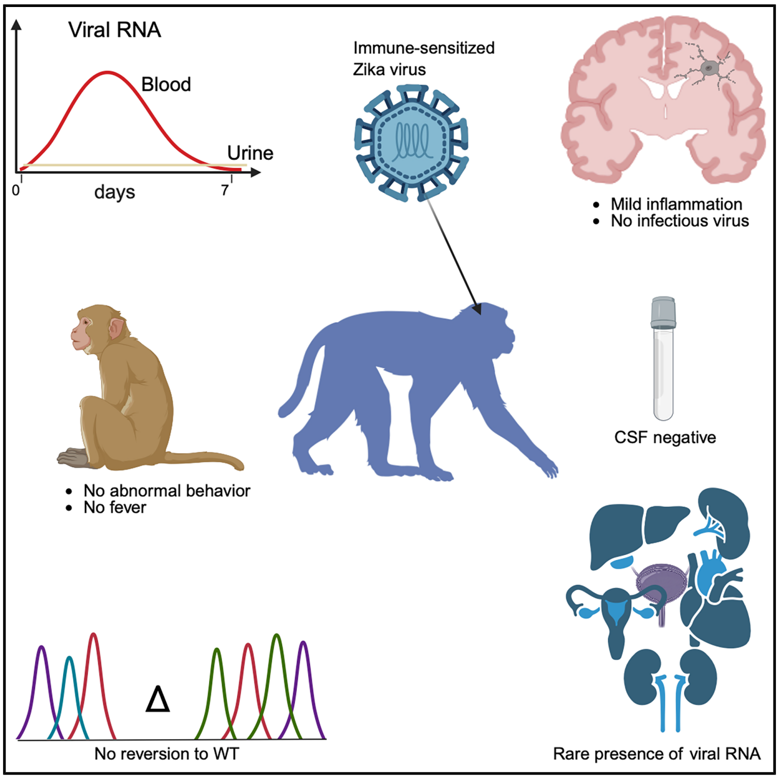

Authors

Alec J. Hirsch,
Amanda de Andrade Costa,
Cody German, ..., Pei-Yong Shi,
Michael S. Diamond, Milan G. Chheda

Correspondence

mchheda@wustl.edu

In brief

Hirsch, Costa, et al. demonstrate that immune-sensitized Zika virus can be safely delivered into primate brains, where it triggers an immune response without causing disease. Their findings suggest potential for a new, safe viral therapy for brain tumors.

Highlights

- Primates intracranially infected with Δ10 3′-UTR ZIKV remained healthy
- Δ10 3′-UTR ZIKV did not revert to WT, and no infectious virus was recovered
- ZIKV RNA was cleared from macaques’ blood within 1 week of intracranial injection
- Brains had mild local inflammation without widespread pathology

Hirsch et al., 2025, Cell Reports Medicine 6, 102509
December 16, 2025 © 2025 The Authors. Published by Elsevier Inc.
https://doi.org/10.1016/j.xcrm.2025.102509

Cell Reports Medicine

CellPress
OPEN ACCESS

Report

Intracranial injection of genetically modified, mosquito non-transmissible Zika virus: Safety in primates and ramifications for brain tumor therapy

Alec J. Hirsch,[1,2,10] Amanda de Andrade Costa,[3,10] Cody German,[1,2] Christopher J. Parkins,[1,2] Jessica L. Smith,[1,2] Emilie Russler-Germain,[3] Ashwani Kesarwani,[3] Yuping Li,[3] Verginia Cuzon Carlson,[7] Timothy Carlson,[7] Jodi L. McBride,[7] Sathya Srinivasan,[1,9] Anne D. Lewis,[6] Xuping Xie,[8] Pei-Yong Shi,[4] Michael S. Diamond,[5] and Milan G. Chheda[3,11,*]

[1]The Vaccine and Gene Therapy Institute, Oregon Health and Sciences University, Beaverton, OR, USA
[2]Division of Pathobiology and Immunology, Oregon National Primate Research Center, Oregon Health and Sciences University, Beaverton, OR, USA
[3]Division of Oncology, Department of Medicine, Washington University School of Medicine, St. Louis, MO, USA
[4]Department of Biochemistry and Molecular Biology, University of Texas Medical Branch, Galveston, TX, USA
[5]Departments of Medicine, Molecular Microbiology, Pathology & Immunology, Washington University School of Medicine, St. Louis, MO, USA
[6]Division of Comparative Medicine, Oregon National Primate Research Center, Oregon Health and Sciences University, Beaverton, OR, USA
[7]Division of Neuroscience, Oregon National Primate Research Center, Oregon Health and Sciences University, Beaverton, OR, USA
[8]Department of Microbiology and Immunology, University of Texas Medical Branch, Galveston, TX, USA
[9]Imaging and Morphology Support Core at the Oregon National Primate Research Center, Beaverton, OR, USA
[10]These authors contributed equally
[11]Lead contact
*Correspondence: mchheda@wustl.edu
https://doi.org/10.1016/j.xcrm.2025.102509

SUMMARY

Glioblastomas (GBMs) are incurable brain tumors. Zika virus (ZIKV) has specificity in killing GBM stem cells, which drive treatment resistance. In mouse models of GBM, ZIKV also generates an anti-tumor inflammatory response and prolongs survival. To support clinical development and address safety concerns for intra-tumoral treatment, we assessed the effects of injection of an immune-sensitized ZIKV (Δ10 3′-UTR ZIKV), which cannot be transmitted by mosquitos, into non-tumor-bearing rhesus macaque brains. After injection, the primates showed no clinical signs of illness. Histologically, as expected, ZIKV infection elicited mild inflammation, which resolved within 2 weeks. No infectious virus was detected in the brain or any organs at 14 dpi. These findings, along with our preclinical observations, support the development of immune-sensitized ZIKV as a treatment for GBM.

INTRODUCTION

Glioblastoma (GBM) is an aggressive brain tumor; median survival is under 21 months.[1] Immune checkpoint inhibitors,[2–4] peptide vaccines,[5,6] and dendritic cell vaccines[7,8] have failed to demonstrate a significant benefit in clinical trials.[9–15] Standard of care has remained mostly unchanged over the last two decades and includes surgery, radiation, chemotherapy, and more recently, anti-mitotic tumor-treating fields.[1] GBM recurrence after maximal treatment often occurs within 6 months and poses a substantial clinical challenge. A highly heterogeneous population of GBM stem cells (GSCs) is resistant to standard therapies and is potentially a driver of recurrence.[16–19] Additionally, the tumor microenvironment is highly immunosuppressive and has a paucity of anti-tumor immune cells.[20–22] We need new therapeutic strategies to address these challenges.

Oncolytic viruses infect and destroy tumor cells and offer potential for GBM treatment.[23] A range of viruses, including H-1 parvovirus,[24] reovirus,[25] measles,[26] Newcastle disease virus,[27] vaccinia virus,[28] poliovirus,[29] adenovirus,[30–32] herpes simplex viruses,[33–35] retroviruses,[36] myxoma virus,[37,38] and vesicular stomatitis virus,[39] have been or are currently being studied as possible therapies for GBM. However, these viruses do not exclusively infect glioma stem cells, and none have thus far been successfully developed for broad clinical use. We and others have shown that oncolytic Zika virus (ZIKV) therapy is unique in that it specifically targets GSCs, while reducing tumor size and extending survival in multiple mouse models of glioma.[40–45] Even though ZIKV causes congenital brain anomalies in fetuses of infected mothers, it rarely infects adult brains.[46] We demonstrated that ZIKV does not infect adult human brain specimens from epilepsy surgery and specifically infects SOX2$^+$ GSCs from human GBM slices.[44] In syngeneic orthotopic murine glioma models, ZIKV therapy alters the microenvironment by increasing immune cell infiltration. Lastly, CD8$^+$ T cells are required for ZIKV-dependent tumor clearance[47] and intra-tumoral oncolytic ZIKV treatment boosts the effect of systemic antibody-mediated PD1 blockade in mice.[47]

© 2025 The Authors. Published by Elsevier Inc.
This is an open access article under the CC BY license (http://creativecommons.org/licenses/by/4.0/).

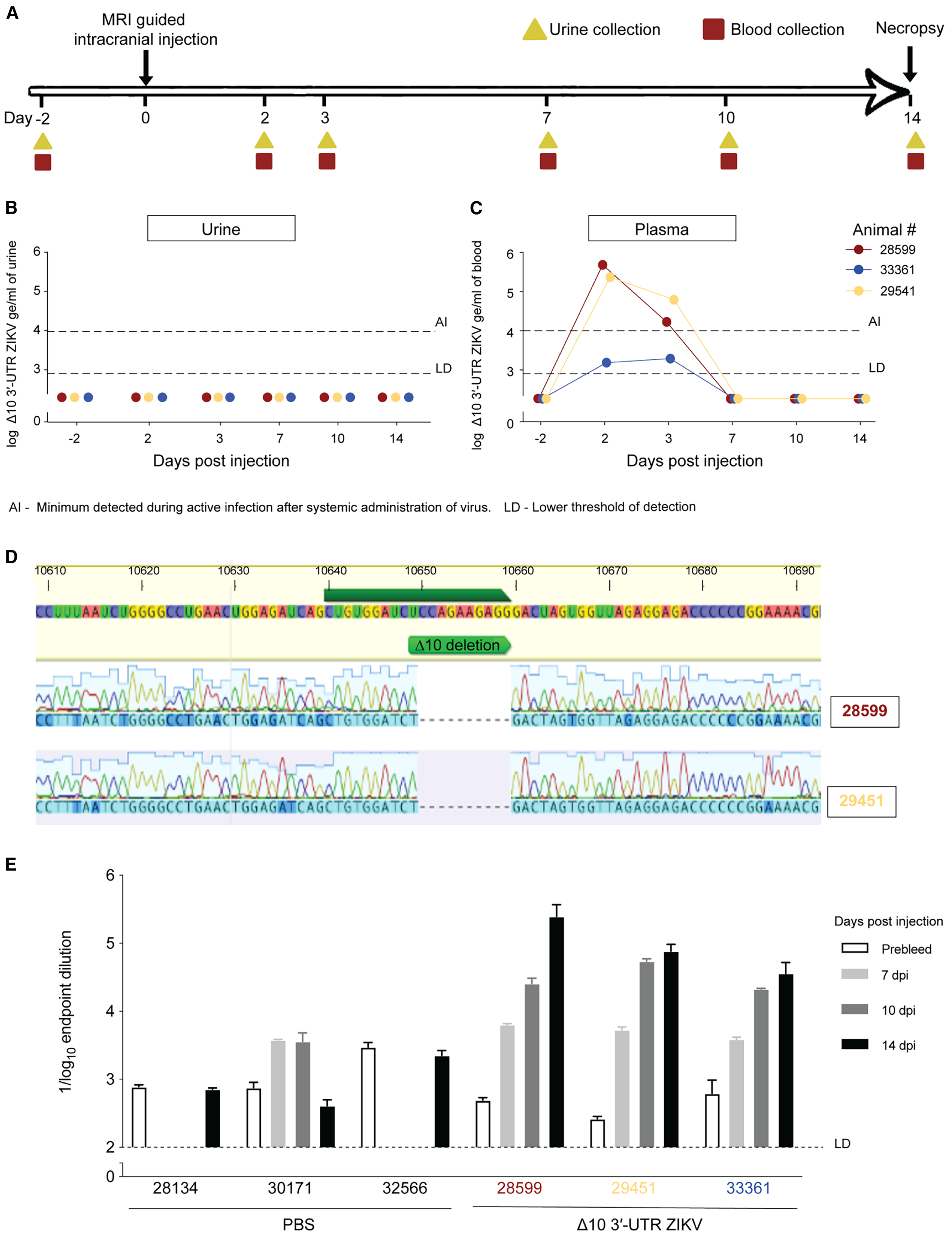

(legend on next page)

For patients, we envision ZIKV as an intra-tumoral therapy administered during surgery. To enhance safety, we engineered an immune-sensitized Δ10 3′-UTR ZIKV by deleting 10 nucleotides in the 3′ untranslated region of the ZIKV-Cambodia clone, eliminating production of the subgenomic flaviviral RNA (sfRNA) species that antagonizes antiviral innate immune responses.[47,48] This virus cannot be transmitted by mosquitoes.[49,50] *In vivo,* Δ10 3′-UTR ZIKV has anti-tumor efficacy and boosts the effects of immune checkpoint blockade.[47]

Murine studies are insufficient to guarantee that Δ10 3′-UTR ZIKV can be safely used in humans. Rhesus macaques are an outstanding non-human primate models to study systemic ZIKV infection, especially in pregnancy model,[50] and the safety studies of ZIKV vaccines.[51,52] We performed a preclinical safety study of Δ10 3′-UTR ZIKV injected into non-tumor-bearing rhesus macaque brains. We monitored animals for 14 days with serial blood and urine sampling and performed postmortem analysis. No neurological signs were observed; we observed a mild brain immune response, and recovered no infectious virus. These findings support the intracranial safety of Δ10 3′-UTR ZIKV and, together with prior data, advance its clinical translation as an oncolytic and immune-modulating therapy for GBM.

RESULTS

Attenuated ZIKV delivered to the brain does not revert to wild-type virus and elicits a humoral immune response

cDNA encoding ZIKV-Cambodia virus was engineered with a deletion of 10 nucleotides in the 3′ UTR of the virus, abrogating production of an sfRNA that antagonizes antiviral immunity in both mosquitoes and mammalian cells.[47] We performed MRI-guided injections in six rhesus macaques, into the white matter of the right frontal cortex (Figure S1). We injected three animals with 100 μL of Δ10 3′-UTR ZIKV (5×10^6 focus-forming units (FFUs)) in PBS; we injected three others with 100 μL of PBS (control group). We predefined a 14 days observational period based on previous work that demonstrated: (1) following subcutaneous inoculation of rhesus macaques with wild-type ZIKV, viral RNA is broadly distributed throughout lymphoid, neuronal, and joint tissues from 7 to 28 dpi[53] and (2) infectious ZIKV is cleared from murine brains 14 days after intracranial injection.[47] In the current study, all animals survived and remained without signs of clinical illness until the time of euthanasia.

Following injection, the animals were observed for behavioral changes, and blood and urine samples were collected 2 days before injection and on 2, 3, 7, 10, and 14 dpi. At day 14 post-injection, the animals were sacrificed (Figure 1A). Using quantitative reverse-transcription PCR (RT-qPCR), we did not detect viral RNA in the urine of any of animals (Figure 1B). ZIKV RNA was detected in the plasma of all three animals from the Δ10 3′-UTR ZIKV group within the first 7 days after injection, peaking at day 2 post-injection (Figure 1C). We performed Sanger sequencing of the region encompassing the Δ10 3′-UTR deletion from plasma viral RNA in two of the three animals (there was not enough genomic material from animal 33361); notably, the 10-nucleotide deletion was maintained (Figure 1D), confirming that, as expected, there was no reversion to wild-type virus.

We next evaluated whether intracranial Δ10 3′-UTR ZIKV induced a humoral response. At 14 dpi, all three Δ10 3′-UTR ZIKV-treated macaques, but not PBS controls, showed ZIKV-reactive immunoglobulin G (IgG), indicating activation of adaptive immunity (Figure 1E), which was required for tumor clearance in our prior mouse studies.[47]

The brains of the macaques injected with Δ10 3′-UTR ZIKV do not have persistent infection

Upon sacrifice, the brains of the six macaques were sectioned proximal to the injection site, into 10 to 12 4-mm thick coronal slices. We took punch biopsies throughout the white matter of the sections as indicated in Figures 2A, S2, and S3. Even-numbered punches were used for infectious virus isolation, and odd-numbered punches for RNA isolation and viral RNA quantification by qRT-PCR. In the examples illustrated in Figures 2A and S2, the location of the punches where traces of viral RNA were detected are highlighted with a blue circle. As expected, we observed traces of ZIKV RNA in some punches, in the vicinity of the injection site in each animal: 5 out of 56, 1 out of 72 and 3 out of 64 punches, respectively (Figure 2B, upper panel). When comparing our findings to previous work where viral RNA concentration was measured at 14 days after systemic ZIKV infection (active infection, AI),[53] we observed that the concentrations of viral RNA collected from the positive brain punches was below those observed during active infection (Figure 2B, lower panel). For 1 punch out of the 3 considered positive for viral RNA in animal 33361, the concentration of genomic material was above the limit of detection for only 1 of 3 technical replicates (Figure 2B lower panel). Finding viral RNA was not surprising, nevertheless, we wanted to rule out the possibility of infectious virus. Homogenates of tissue samples adjacent to viral RNA positive samples were incubated with C6/36 cells for 7 days, followed by incubation of culture supernatant on Vero cells for 3 days, as

Figure 1. ZIKV RNA is cleared from the plasma within a week of intracranial injection in rhesus macaques

(A) Timeline of experiments. Under MRI guidance, rhesus macaques were intracranially injected with 5×10^6 focus-forming units (FFUs) of Δ10 3′-UTR ZIKV in 100 μL of PBS ($n = 3$) or 100 μL of PBS alone ($n = 3$). Blood and urine from the animals were collected 2 days before injection and at 2, 3, 7, 10, and 14 dpi. At 14 dpi, the animals were necropsied.

(B) Viral RNA levels in urine.

(C) Viral RNA levels in plasma. Dashed line LD marks the lower limit of detection of the assay, and dashed line AI shows minimum levels that have been previously observed during active systemic infection.[53] All analyses were performed using three technical replicates per sample.

(D) Viral RNA was extracted from the plasma of the infected animals, and the region containing the 10-nucleotide deletion was amplified. Sanger sequencing of the amplicon confirmed persistence of the 10-nucleotide deletion. We were unable to amplify this region from animal 33,361, which had lower levels of viral RNA in the plasma.

(E) ELISA results comparing plasma concentrations of anti-Δ10 3′-UTR ZIKV-reactive IgG between 2 days before intracranial injection of Δ10 3′-UTR ZIKV or PBS and 7, 10, and 14 dpi. Error bars (SEM) represent the variation in three technical replicates.

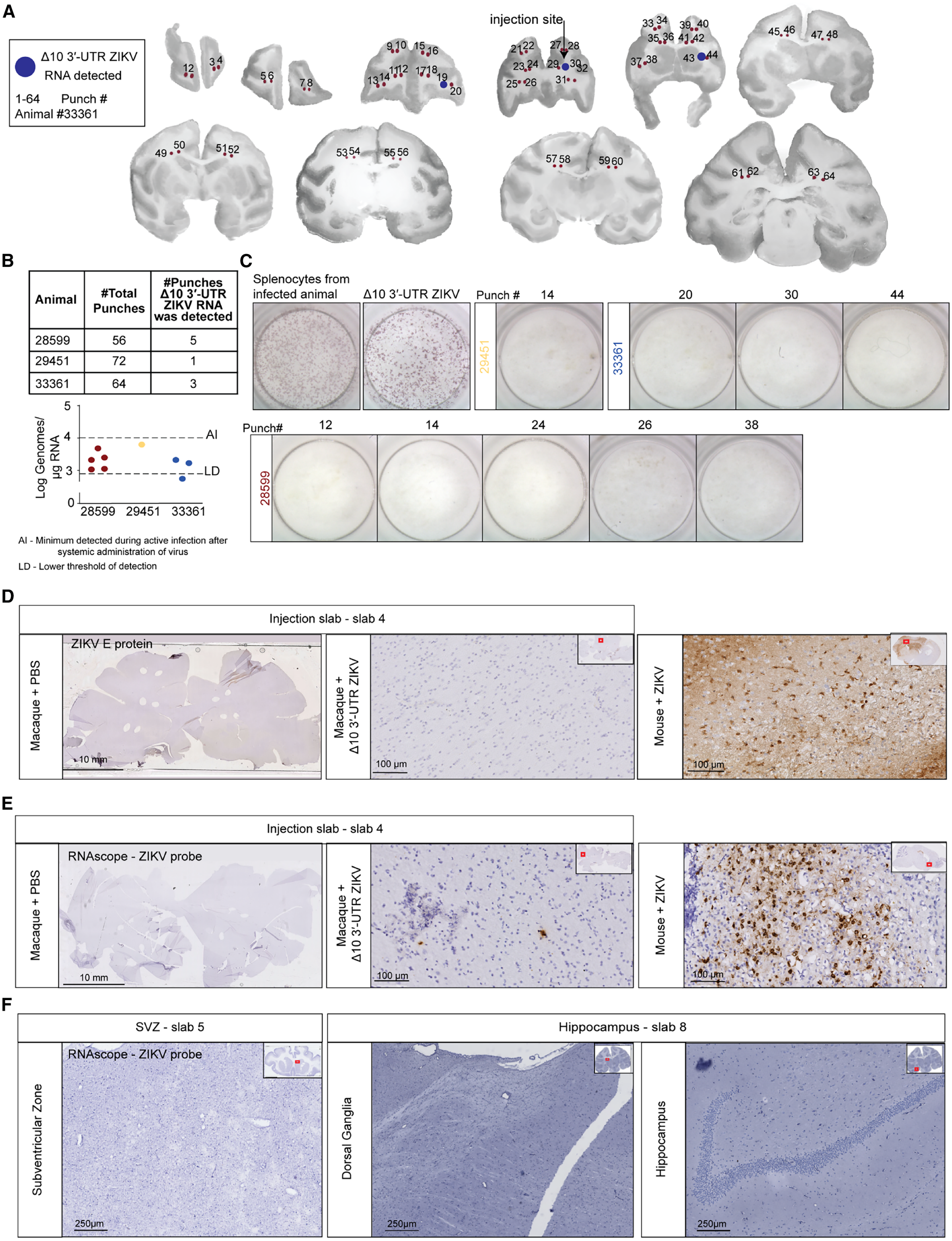

(legend on next page)

CellPress
OPEN ACCESS

described.[49,53] The Vero cells were fixed and stained using an antibody against ZIKV envelope (E) protein, and revealed no evidence of viral antigen, demonstrating absence of infectious virus. We used two separate positive controls: Vero cells infected *in vitro* directly with 10^8 FFUs of Δ10 3′-UTR ZIKV and splenocytes isolated at 5 dpi from a separate, rhesus macaque that was inoculated subcutaneously on the lower arms as previously described[53] with wild-type ZIKV (Dakar 41524). We detected live virus in Vero cells exposed to the supernatants of the splenocytes but not from any brain (Figure 2C).

To confirm and expand our findings, tissue blocks with detectable viral RNA were sectioned (20 μm) and analyzed by immunohistochemistry for ZIKV E protein and *in situ* hybridization for ZIKV RNA. Wild-type ZIKV-infected, tumor-bearing mouse brains served as positive controls. In all macaque brains, E protein staining was not detected (Figure 2D), and only sparse ZIKV RNA was detected on the ipsilateral side of Δ10 3′-UTR ZIKV-injected macaques, but not in PBS controls (Figure 2E). We did not find ZIKV RNA in areas where neural stem cells reside in the adult brain (subventricular zone, basal ganglia and hippocampus, Figure 2F). Together, these findings suggest that by 14 days after infection, there was no longer infectious virus in macaque brains.

Δ10 3′-UTR ZIKV treatment is associated with activated microglia

Our previous murine GBM studies showed that ZIKV kills tumor cells and enhances anti-tumor immunity.[47] We therefore hypothesized that intracranial ZIKV injection in macaques would elicit a moderate immune response in the absence of tumors. To assess inflammation, we performed hematoxylin and eosin staining on brain sections. Signs of mild inflammation were apparent on the ipsilateral injection site in all three of the Δ10 3′-UTR ZIKV-injected brains, including lymphocytic infiltration and perivascular cuffing in white matter that extended to the gray matter, activated microglia, mild gliosis, and edema (Figure 3A) but not in the PBS injected macaques.

Staining with an antibody against CD3 (Figure 3B) did not reveal a significant difference in infiltration of T cells between PBS and Δ10 3′-UTR ZIKV-injected brains (Figure 3C). Staining with an antibody against IBA1, a marker of microglia and monocyte-derived macrophages (Figure 3D), demonstrated focal areas populated with ameboid-like, activated microglia in the Δ10 3′-UTR ZIKV-challenged brains that were not present in the PBS-treated brains (Figure S4A); with no statistically significant difference, possibly because of the small sample size (Figure 3E). Also, there was no statistically significant difference in density of CCR2-expressing monocytes or CD11c-expressing dendritic cells (Figures S4B and S4C). Finally, using a murine model of glioma followed by intracranial injection of Δ10 3′-UTR ZIKV, we determined that the presence of ZIKV causes a significantly higher infiltration of CD8-expressing cells in the tumor in comparison to PBS (Figure S4D), supporting the idea that tumor-associated inflammation plays a critical role in driving T cell infiltration, consistent with our prior work.[47]

Viral RNA is largely absent in peripheral organs from the Δ10 3′-UTR ZIKV-injected macaques

At 14 dpi, cerebrospinal fluid and tissues from multiple organs were collected for viral RNA quantification (Figure 4). The detection limit was 100 genome copies per 0.2 μg RNA. For comparison, RNA levels from macaques with active systemic ZIKV infection (AI) are shown based on prior data.[53] Viral RNA was not detected in the cerebrospinal fluid, sciatic nerve, trigeminal ganglion, cervical, thoracic, or lumbar spinal cord, eye, elbows, kidney, bladder, vagina, cervix, heart, colon, ileum, duodenum, jejunum, liver, or lymphoid tissues (spleen, bone marrow, cervical, inguinal, mesenteric, or axillary lymph nodes) in any of the 3 animals injected with Δ10 3′-UTR ZIKV at 14 dpi (Figures 4A–4C and 4E–4G). At sacrifice, animal 28599 had detectable viral RNA in the brachial plexus and uterus, animals 28599 and 29451 had ZIKV RNA present in knee tissues (Figure 4D), and animal 28599 had measurable ZIKV RNA in the stomach (Figure 4G).

DISCUSSION

Oncolytic viruses are wild-type or genetically modified viruses that kill cancer cells. There are several oncolytic viruses under study for treatment of GBM, including four herpes simplex viruses (rQNestin34.5v.2, NCT03152318; C134, NCT03657576; M032, NCT02062827 and NCT05084430; and HSV G207Δ, NCT03911388) and two adenoviruses (Delta-24-RGD-DNX-2401, NCT03896568; ICOVIR-5, NCT04758533). None have

Figure 2. Viral RNA and infectious virus levels in rhesus macaque brains 14 days after intracranial injection of Δ10 3′-UTR ZIKV

(A) After perfusion with PBS, the brain was cut into 4 mm coronal slabs, and 2 mm diameter punches from both hemispheres were collected for detection of viral RNA and infectious virus. The illustration is based on pictures taken of the slabs using Photopea software and depicts the location of the punches extracted from one of the animals (animal number 33,361). Blue dots show the punches where viral RNA was found.

(B) Odd-numbered punches were used for measuring ZIKV RNA (upper panel). Quantification of the concentration of viral RNA in 3 technical replicates per brain punch (lower panel).

(C) Even-numbered punches were homogenized and added to C6/36 cells. After 7 days, 50 μL of culture supernatant was transferred to plated Vero cells. Virus was detected by focus formation assay with an immunocytostaining for ZIKV E protein.[53] For comparison, splenocytes from a wild-type ZIKV (strain Dakar 41524) systemically infected animal were similarly cultured with C6/36 and assayed on Vero cells ("Splenocytes from ZIKV-infected animal"). ZIKV Δ10 3′-UTR was also added directly to Vero cells ("ZIKVΔ10").

(D) ZIKV E protein was also detected in mice infected with WT ZIKV ($n = 3$) but not Δ10 3′-UTR ZIKV ($n = 3$)- or PBS ($n = 3$)-injected macaque brains, representative picture from the brain of 28,599 (ZIKV treated) and 30,171(PBS treated). Scale bars: 10 mm (left) and 100 μm (central and right).

(E) Scattered viral RNA staining found by *in situ* hybridization of brains of Δ10 3′-UTR ZIKV ($n = 3$)- but not PBS ($n = 3$)-injected macaques at 14 dpi, representative picture from the brain of 28,599 (ZIKV treated) and 30,171(PBS treated). Mouse brains implanted with SB28 tumor cells and injected with mouse-adapted ZIKV (10^5 FFUs, $n = 3$) were used as positive staining controls for experiments depicted in (D) and (E). Scale bars: 10 mm (left) and 100 μm (central and right).

(F) Viral RNA not found by *in situ* hybridization of subventricular zone, basal ganglia, and hippocampus of Δ10 3′-UTR ZIKV-injected macaques at 14 dpi, representative picture from the brain of 28,599 (ZIKV treated). Scale bar: 250 μm.

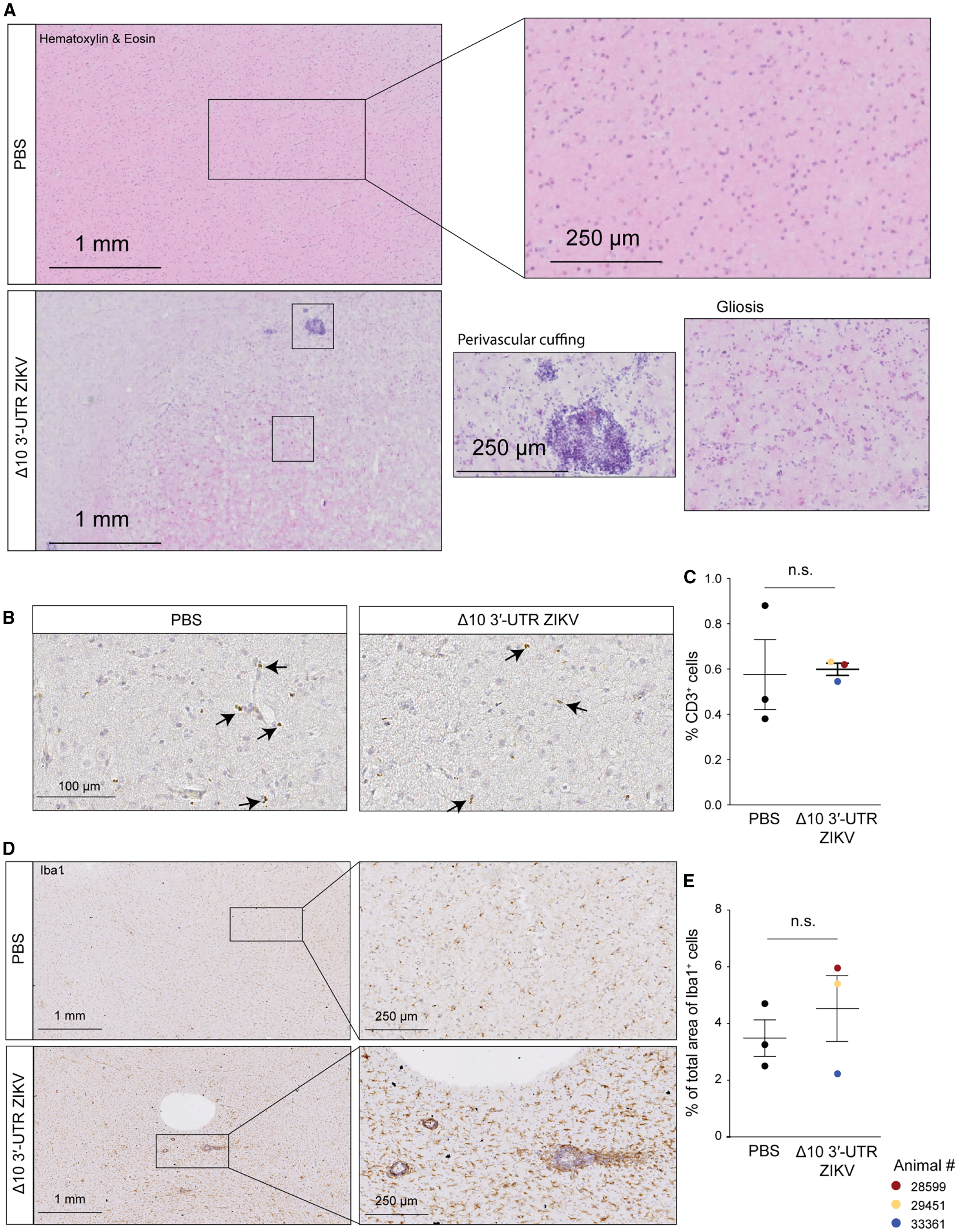

(legend on next page)

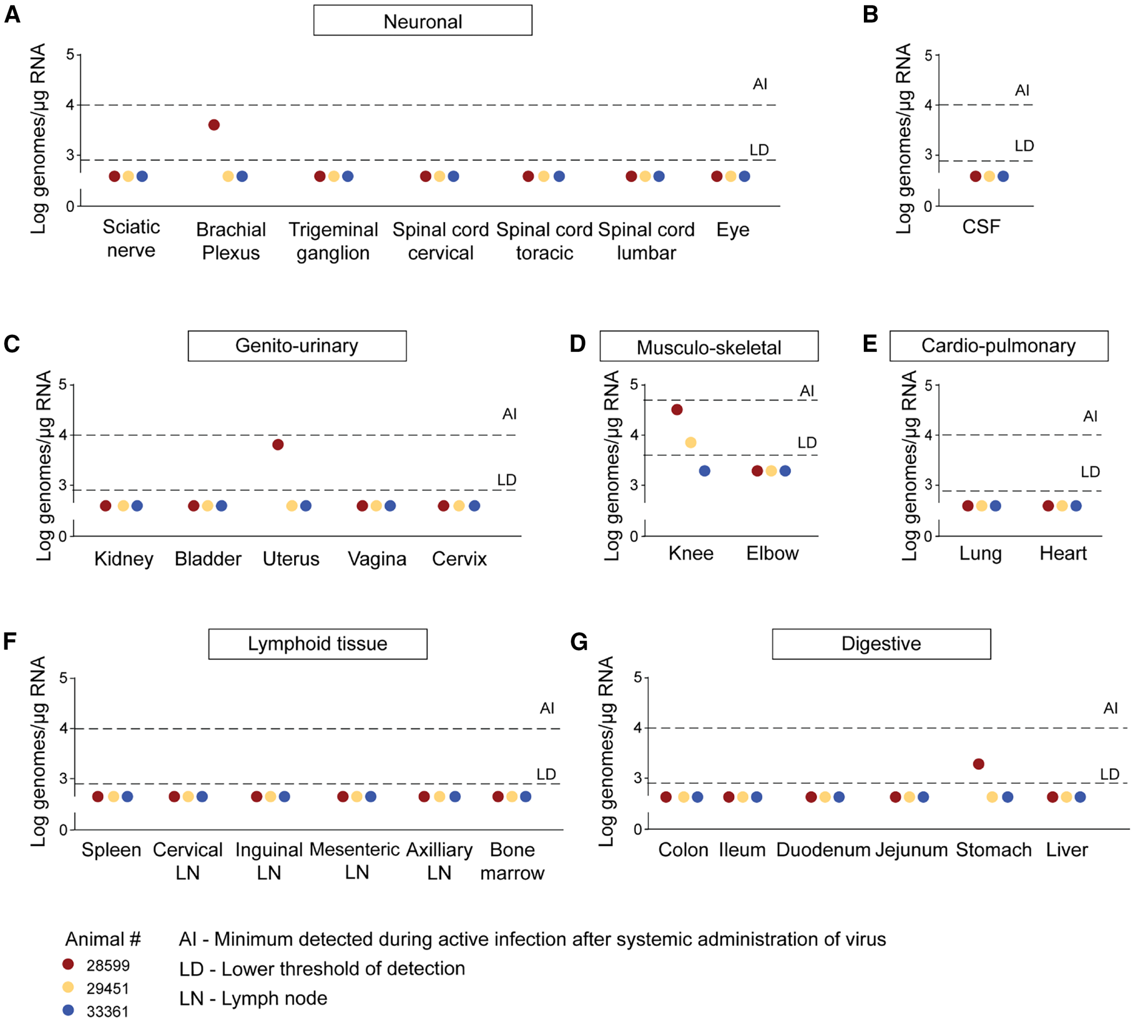

Figure 4. ZIKV RNA was not detectable in most organs at 14 dpi

(A) Viral levels of RNA in the sciatic nerve, brachial plexus, trigeminal ganglion, spinal cord—cervical, thoracic, and lumbar, and the eye.
(B) Viral RNA levels in the cerebrospinal fluid of the Δ10 3′-UTR ZIKV-injected animals (*n* = 3).
(C) Levels of ZIKV RNA in the kidneys, bladder, uterus, vagina, or cervix of the three animals injected with Δ10 3′-UTR ZIKV.
(D) Viral RNA levels in the knee and elbows the animals.
(E) Levels of ZIKV RNA in the lungs and hearts of the animals in this study.
(F) Viral RNA levels in the spleens, cervical, inguinal, mesenteric, axillary lymph nodes, and the bone marrow of the animals injected with Δ10 3′-UTR ZIKV.
(G) Viral RNA levels in the colon, ileum, duodenum, jejunum, stomach, and liver of the animals.

yet been approved by the U.S. Food and Drug Administration (FDA). Genetically engineered herpes virus has received conditional and time-limited approval in Japan, based on a single arm phase 2 trial in patients with progressive GBM.[54] Talimogene laherparepvec (T-VEC), a genetically modified herpes virus to treat metastatic melanoma is the only FDA-approved oncolytic therapy.[55] Our previous studies demonstrated efficacy of Δ10 3′-UTR ZIKV in treating murine models of high-grade glioma.[47] Δ10 3′-UTR ZIKV is effective against the GL261 glioma model as a single agent, and against CT2A glioma when combined with anti-PD1 immunotherapy.[47] Analysis of the kinetics of viral replication in the mouse brain demonstrated that 2 weeks

Figure 3. Mild inflammation in the brains of Δ10 3′-UTR ZIKV-injected macaques

(A) Hematoxylin and eosin staining of brains from Δ10 3′-UTR ZIKV (*n* = 3)- and PBS (*n* = 3)-injected macaques at 14 dpi. Scale bar: 1 mm (left) and 250 µm (right).
(B and C) Staining (B) and quantification (C) of CD3-positive T cells in the brains of PBS- and Δ10 3′-UTR ZIKV-injected macaques. Scale bar: 100 µm.
(D and E) Staining (D) and quantification (E) of the total area of tissue stained with anti-IBA1 antibody (microglia- and monocyte-derived macrophages) normalized to the total area of brain tissue in brain sections of PBS and Δ10 3′-UTR ZIKV-injected macaques.
n.s., non significant

after treatment, ZIKV RNA remained confined to the tumor and that clearance of infectious ZIKV happens by 14 dpi.[44,47]

Rhesus macaques recapitulate several clinical and virological features of systemic ZIKV infection in humans and thus are good models to study ZIKV infection, pathogenesis, and vaccine candidates.[50,52] Intracranial delivery of immune-sensitized ZIKV has not been previously tested. Because Δ10 3′-UTR ZIKV is intended for intraoperative use in GBM patients, we evaluated its safety after intracranial injection in rhesus macaques by analyzing blood, urine, and tissues for viral RNA and infectious virus. Infectious Δ10 3′-UTR ZIKV was not detected in any samples tested, and plasma RNA cleared within 7 days. Brain tissue showed no viral antigen or RNA, and only mild, nonsignificant inflammation compared with PBS controls. Mice bearing gliomas treated with ZIKV, had tumor cell death, followed by T cell infiltration into the tumor microenvironment (Figure S4D). To our knowledge, GBM models in non-human primates do not exist, thus this study only assessed ZIKV effects in non-tumor-bearing brains. Consequently, the inflammation observed differs in magnitude and composition from that in tumor-bearing mice. Reassuringly, the macaques remained without clinical signs of illness with no neurological deficits.

We compared ZIKV RNA concentrations in organs in our study with concentrations previously reported after systemic infection when animals were displaying active infection symptoms. While we detected viral RNA at 14 dpi in a few organs in a few animals, levels were lower than that measured during active infection.[53] Our findings were similar to other studies in which non-human primates were used to assess safety viruses currently in clinical trials, namely, absence of significant pathology caused by the intracranially injected virus, increase in blood immunoglobulin levels, absence of evidence of viral dissemination, and signs of local mild inflammation in the brain.[56–58]

One purpose of this study is to inform intra-tumoral dosing for clinical trials in brain tumor patients. ZIKV targets cancer stem cells and induces $CD8^+$ T cell infiltration and subsequent tumor clearance.[47] After intracranial injection of 5×10^6 FFUs Δ10 3′-UTR ZIKV, we observed viral clearance and a mild immune response. This dose was well tolerated in primates, with no neurological or pathological changes up to 2 weeks post-infection. Increased ZIKV-reactive IgG levels indicated antigen trafficking. Overall, Δ10 3′-UTR ZIKV appears safe for intracranial administration without evidence of encephalitis.

This study suggests safety of the Δ10 3′-UTR ZIKV. The 3′UTR deletion eliminates sfRNA production, making the virus non–mosquito-transmissible and minimizing environmental risk. We observed no reversion to wild-type status *in vivo*. Still, translation into the clinic will require careful dose escalation studies to balance anti-tumor efficacy with potential toxicity.

We envision intraoperative administration of Δ10 3′-UTR ZIKV. Patients will need close post-surgical monitoring with imaging or biopsy to track viral activity, tumor response, and immune infiltration. Clinical translation will require regulatory approval supported by safety. Patient immune status and prior therapies must be considered, and combinations with checkpoint inhibitors, vaccines, or immune-stimulating agents may enhance efficacy.

In summary, our results, combined with prior work in mouse models demonstrating efficacy, support the continued development of Δ10 3′-UTR ZIKV as a treatment for brain tumors. While challenges remain in optimizing dosing and patient selection, these early safety findings establish a foundation for advancing ZIKV into human clinical trials.

Limitations of the study

There are several limitations to this study. First, the small number of rhesus macaques reduces statistical power and limits the ability to detect subtle effects. Second, the experiments were conducted in non-tumor-bearing animals, which may not fully replicate the physiological and immunologic context of a tumor-bearing host. Finally, only a single viral dose (5×10^6 FFUs) was evaluated, preventing assessment of a dose-response relationship or determination of a maximum tolerated dose. It is possible that higher or repeated doses could lead to different safety or immunologic outcomes.

RESOURCE AVAILABILITY

Lead contact

Further information and requests for resources and reagents should be directed to and will be fulfilled by the lead contact, Milan G. Chheda (mchheda@wustl.edu).

Materials availability

This study did not generate new unique reagents.

Data and code availability

All data reported in this paper will be shared by the lead contact upon request. This paper does not report original code. Any additional information required to reanalyze the data reported in this paper is available from the lead contact upon request.

ACKNOWLEDGMENTS

Research reported in this publication was supported by the Robert J. Kleberg, Jr. and Helen C. Kleberg Foundation and NINDS/NIH under award numbers R01NS107833 and R01NS117149 (M.G.C.) and Cancer Biology Pathway/Molecular Oncology T32 Program (A.d.A.C.). This research was supported by the Alvin J. Siteman Cancer Center through The Foundation for Barnes-Jewish Hospital. We also acknowledge support from the Hope Center for Neurological Disorders (Shared Instrumentation Grant - NCRR 1S10RR027552). Illustrations were created on BioRender.

AUTHOR CONTRIBUTIONS

A.d.A.C., formal writing (original draft and review and editing), experiments, and analysis. A.J.H., formal writing (review and editing), experiments, and analysis. A.J.H., C.G., C.J.P., J.L.S., A.K., Y.L., V.C.C., T.C., J.L.M., and S.S., experiments and analysis. A.D.L., and E.R-G., pathologists-evaluated H&E staining and IHC/RNAscope images, respectively. X.X., conceptualization and provision of the virus. P.-Y.S., conceptualization and provision of the virus. M.S.D., conceptualization, investigation, writing (review and editing), project administration, and funding acquisition. M.G.C., conceptualization, investigation, writing (original draft and review and editing), supervision, project administration, and funding acquisition.

DECLARATION OF INTERESTS

M.S.D. is a consultant or advisor for InBios, IntegerBio, Akagera Medicines, Merck, GlaxoSmithKline, and Moderna. The Diamond laboratory has received unrelated funding support in sponsored research agreements from Moderna.

CellPress
OPEN ACCESS

M.S.D., P.-Y.S., and M.G.C. hold patents for use of Δ10 3′-UTR ZIKV to treat brain tumors. M.G.C. receives royalties from UpToDate and has received funding for clinical trials and/or laboratory research from Merck, Orbus Therapeutics, Incyte, and NeoimmuneTech.

STAR★METHODS

Detailed methods are provided in the online version of this paper and include the following:

- KEY RESOURCES TABLE
- EXPERIMENTAL MODEL AND SUBJECT DETAILS
  - Ethics statement regarding non-human primate research
  - Virus
  - Mouse brains
  - Non-human primates
- METHOD DETAILS
  - Magnetic resonance imaging (MRI)-guided stereotactic surgery
  - Preparation of NHP brain sections
- QUANTIFICATION AND STATISTICAL ANALYSIS
  - Immunohistochemistry
  - Detection of infectious virus in brain
  - Quantification of viral RNA
  - Viral RNA *in situ* hybridization
  - ELISA
  - Statistical analysis

SUPPLEMENTAL INFORMATION

Supplemental information can be found online at https://doi.org/10.1016/j.xcrm.2025.102509.

Received: October 8, 2024
Revised: September 8, 2025
Accepted: November 14, 2025
Published: December 16, 2025

REFERENCES

1. Stupp, R., Taillibert, S., Kanner, A., Read, W., Steinberg, D., Lhermitte, B., Toms, S., Idbaih, A., Ahluwalia, M.S., Fink, K., et al. (2017). Effect of Tumor-Treating Fields Plus Maintenance Temozolomide vs Maintenance Temozolomide Alone on Survival in Patients With Glioblastoma: A Randomized Clinical Trial. JAMA *318*, 2306–2316. https://doi.org/10.1001/jama.2017.18718.
2. Ferris, R.L., Blumenschein, G., Fayette, J., Guigay, J., Colevas, A.D., Licitra, L., Harrington, K., Kasper, S., Vokes, E.E., Even, C., et al. (2016). Nivolumab for Recurrent Squamous-Cell Carcinoma of the Head and Neck. N. Engl. J. Med. *375*, 1856–1867. https://doi.org/10.1056/NEJMoa1602252.
3. Wolchok, J.D., Kluger, H., Callahan, M.K., Postow, M.A., Rizvi, N.A., Lesokhin, A.M., Segal, N.H., Ariyan, C.E., Gordon, R.-A., Reed, K., et al. (2013). Nivolumab plus Ipilimumab in Advanced Melanoma. N. Engl. J. Med. *369*, 122–133. https://doi.org/10.1056/NEJMoa1302369.
4. Jia, W., Jackson-Cook, C., and Graf, M.R. (2010). Tumor-infiltrating, myeloid-derived suppressor cells inhibit T cell activity by nitric oxide production in an intracranial rat glioma+vaccination model. J. Neuroimmunol. *223*, 20–30. https://doi.org/10.1016/j.jneuroim.2010.03.011.
5. Naghavian, R., Faigle, W., Oldrati, P., Wang, J., Toussaint, N.C., Qiu, Y., Medici, G., Wacker, M., Freudenmann, L.K., Bonté, P.E., et al. (2023). Microbial peptides activate tumour-infiltrating lymphocytes in glioblastoma. Nature *617*, 807–817. https://doi.org/10.1038/s41586-023-06081-w.
6. Swartz, A.M., Batich, K.A., Fecci, P.E., and Sampson, J.H. (2015). Peptide vaccines for the treatment of glioblastoma. J. Neuro Oncol. *123*, 433–440. https://doi.org/10.1007/s11060-014-1676-y.
7. Zhou, J., Li, L., Jia, M., Liao, Q., Peng, G., Luo, G., and Zhou, Y. (2023). Dendritic cell vaccines improve the glioma microenvironment: Influence, challenges, and future directions. Cancer Med. *12*, 7207–7221. https://doi.org/10.1002/cam4.5511.
8. Zhu, P., Li, S.Y., Ding, J., Fei, Z., Sun, S.N., Zheng, Z.H., Wei, D., Jiang, J., Miao, J.L., Li, S.Z., et al. (2023). Combination immunotherapy of glioblastoma with dendritic cell cancer vaccines, anti-PD-1 and poly I:C. J. Pharm. Anal. *13*, 616–624. https://doi.org/10.1016/j.jpha.2023.04.012.
9. McGranahan, T., Therkelsen, K.E., Ahmad, S., and Nagpal, S. (2019). Current State of Immunotherapy for Treatment of Glioblastoma. Curr. Treat. Options Oncol. *20*, 24. https://doi.org/10.1007/s11864-019-0619-4.
10. Preusser, M., Lim, M., Hafler, D.A., Reardon, D.A., and Sampson, J.H. (2015). Prospects of immune checkpoint modulators in the treatment of glioblastoma. Nat. Rev. Neurol. *11*, 504–514. https://doi.org/10.1038/nrneurol.2015.139.
11. Hilf, N., Kuttruff-Coqui, S., Frenzel, K., Bukur, V., Stevanović, S., Gouttefangeas, C., Platten, M., Tabatabai, G., Dutoit, V., van der Burg, S.H., et al. (2019). Actively personalized vaccination trial for newly diagnosed glioblastoma. Nature *565*, 240–245. https://doi.org/10.1038/s41586-018-0810-y.
12. Ahluwalia, M.S., Reardon, D.A., Abad, A.P., Curry, W.T., Wong, E.T., Figel, S.A., Mechtler, L.L., Peereboom, D.M., Hutson, A.D., Withers, H.G., et al. (2023). Phase IIa Study of SurVaxM Plus Adjuvant Temozolomide for Newly Diagnosed Glioblastoma. J. Clin. Oncol. *41*, 1453–1465. https://doi.org/10.1200/jco.22.00996.
13. Hotchkiss, K.M., Batich, K.A., Mohan, A., Rahman, R., Piantadosi, S., and Khasraw, M. (2023). Dendritic cell vaccine trials in gliomas: Untangling the lines. Neuro Oncol. *25*, 1752–1762. https://doi.org/10.1093/neuonc/noad088.
14. Wang, Y., Li, S., Peng, Y., Ma, W., Wang, Y., and Li, W. (2023). Progress in phase III clinical trials of molecular targeted therapy and immunotherapy for glioblastoma. Cancer Innov. *2*, 114–130. https://doi.org/10.1002/cai2.59.
15. Lim, M., Weller, M., Idbaih, A., Steinbach, J., Finocchiaro, G., Raval, R.R., Ansstas, G., Baehring, J., Taylor, J.W., Honnorat, J., et al. (2022). Phase III trial of chemoradiotherapy with temozolomide plus nivolumab or placebo for newly diagnosed glioblastoma with methylated MGMT promoter. Neuro Oncol. *24*, 1935–1949. https://doi.org/10.1093/neuonc/noac116.
16. Gimple, R.C., Bhargava, S., Dixit, D., and Rich, J.N. (2019). Glioblastoma stem cells: lessons from the tumor hierarchy in a lethal cancer. Genes Dev. *33*, 591–609. https://doi.org/10.1101/gad.324301.119.
17. Bao, S., Wu, Q., McLendon, R.E., Hao, Y., Shi, Q., Hjelmeland, A.B., Dewhirst, M.W., Bigner, D.D., and Rich, J.N. (2006). Glioma stem cells promote radioresistance by preferential activation of the DNA damage response. Nature *444*, 756–760. https://doi.org/10.1038/nature05236.
18. Xie, X.P., Laks, D.R., Sun, D., Ganbold, M., Wang, Z., Pedraza, A.M., Bale, T., Tabar, V., Brennan, C., Zhou, X., and Parada, L.F. (2022). Quiescent human glioblastoma cancer stem cells drive tumor initiation, expansion, and recurrence following chemotherapy. Dev. Cell *57*, 32–46.e8. https://doi.org/10.1016/j.devcel.2021.12.007.
19. Lauko, A., Volovetz, J., Turaga, S.M., Bayik, D., Silver, D.J., Mitchell, K., Mulkearns-Hubert, E.E., Watson, D.C., Desai, K., Midha, M., et al. (2022). SerpinB3 drives cancer stem cell survival in glioblastoma. Cell Rep. *40*, 111348. https://doi.org/10.1016/j.celrep.2022.111348.
20. El Andaloussi, A., and Lesniak, M.S. (2006). An increase in CD4+CD25+FOXP3+ regulatory T cells in tumor-infiltrating lymphocytes of human glioblastoma multiforme. Neuro Oncol. *8*, 234–243. https://doi.org/10.1215/15228517-2006-006.
21. Han, S., Zhang, C., Li, Q., Dong, J., Liu, Y., Huang, Y., Jiang, T., and Wu, A. (2014). Tumour-infiltrating CD4(+) and CD8(+) lymphocytes as predictors of clinical outcome in glioma. Br. J. Cancer *110*, 2560–2568. https://doi.org/10.1038/bjc.2014.162.

22. Raychaudhuri, B., Rayman, P., Huang, P., Grabowski, M., Hambardzumyan, D., Finke, J.H., and Vogelbaum, M.A. (2015). Myeloid derived suppressor cell infiltration of murine and human gliomas is associated with reduction of tumor infiltrating lymphocytes. J. Neuro Oncol. *122*, 293–301. https://doi.org/10.1007/s11060-015-1720-6.

23. Qi, Z., Long, X., Liu, J., and Cheng, P. (2022). Glioblastoma microenvironment and its reprogramming by oncolytic virotherapy. Front. Cell. Neurosci. *16*, 819363. https://doi.org/10.3389/fncel.2022.819363.

24. Angelova, A.L., Barf, M., Geletneky, K., Unterberg, A., and Rommelaere, J. (2017). Immunotherapeutic Potential of Oncolytic H-1 Parvovirus: Hints of Glioblastoma Microenvironment Conversion towards Immunogenicity. Viruses *9*. Viruses *9*, 382. https://doi.org/10.3390/v9120382.

25. Samson, A., Scott, K.J., Taggart, D., West, E.J., Wilson, E., Nuovo, G.J., Thomson, S., Corns, R., Mathew, R.K., Fuller, M.J., et al. (2018). Intravenous delivery of oncolytic reovirus to brain tumor patients immunologically primes for subsequent checkpoint blockade. Sci. Transl. Med. *10*, eaam7577. https://doi.org/10.1126/scitranslmed.aam7577.

26. Allen, C., Vongpunsawad, S., Nakamura, T., James, C.D., Schroeder, M., Cattaneo, R., Giannini, C., Krempski, J., Peng, K.W., Goble, J.M., et al. (2006). Retargeted oncolytic measles strains entering via the EGFRvIII receptor maintain significant antitumor activity against gliomas with increased tumor specificity. Cancer Res. *66*, 11840–11850. https://doi.org/10.1158/0008-5472.Can-06-1200.

27. Fan, X., Lu, H., Cui, Y., Hou, X., Huang, C., and Liu, G. (2018). Overexpression of p53 delivered using recombinant NDV induces apoptosis in glioma cells by regulating the apoptotic signaling pathway. Exp. Ther. Med. *15*, 4522–4530. https://doi.org/10.3892/etm.2018.5935.

28. Vasileva, N., Ageenko, A., Dmitrieva, M., Nushtaeva, A., Mishinov, S., Kochneva, G., Richter, V., and Kuligina, E. (2021). Double Recombinant Vaccinia Virus: A Candidate Drug against Human Glioblastoma. Life *11*, 1084. https://doi.org/10.3390/life11101084.

29. Desjardins, A., Gromeier, M., Herndon, J.E., Beaubier, N., Bolognesi, D.P., Friedman, A.H., Friedman, H.S., McSherry, F., Muscat, A.M., Nair, S., et al. (2018). Recurrent Glioblastoma Treated with Recombinant Poliovirus. N. Engl. J. Med. *379*, 150–161. https://doi.org/10.1056/NEJMoa1716435.

30. Huang, J., Zheng, M., Zhang, Z., Tang, X., Chen, Y., Peng, A., Peng, X., Tong, A., and Zhou, L. (2021). Interleukin-7-loaded oncolytic adenovirus improves CAR-T cell therapy for glioblastoma. Cancer Immunol. Immunother. *70*, 2453–2465. https://doi.org/10.1007/s00262-021-02856-0.

31. Ito, H., Aoki, H., Kühnel, F., Kondo, Y., Kubicka, S., Wirth, T., Iwado, E., Iwamaru, A., Fujiwara, K., Hess, K.R., et al. (2006). Autophagic cell death of malignant glioma cells induced by a conditionally replicating adenovirus. J. Natl. Cancer Inst. *98*, 625–636. https://doi.org/10.1093/jnci/djj161.

32. Jiang, H., McCormick, F., Lang, F.F., Gomez-Manzano, C., and Fueyo, J. (2006). Oncolytic adenoviruses as antiglioma agents. Expert Rev. Anticancer Ther. *6*, 697–708. https://doi.org/10.1586/14737140.6.5.697.

33. Jahan, N., Lee, J.M., Shah, K., and Wakimoto, H. (2017). Therapeutic targeting of chemoresistant and recurrent glioblastoma stem cells with a proapoptotic variant of oncolytic herpes simplex virus. Int. J. Cancer *141*, 1671–1681. https://doi.org/10.1002/ijc.30811.

34. Kanai, R., Wakimoto, H., Martuza, R.L., and Rabkin, S.D. (2011). A novel oncolytic herpes simplex virus that synergizes with phosphoinositide 3-kinase/Akt pathway inhibitors to target glioblastoma stem cells. Clin. Cancer Res. *17*, 3686–3696. https://doi.org/10.1158/1078-0432.Ccr-10-3142.

35. Mineta, T., Rabkin, S.D., Yazaki, T., Hunter, W.D., and Martuza, R.L. (1995). Attenuated multi-mutated herpes simplex virus-1 for the treatment of malignant gliomas. Nat. Med. *1*, 938–943. https://doi.org/10.1038/nm0995-938.

36. Perez, O.D., Logg, C.R., Hiraoka, K., Diago, O., Burnett, R., Inagaki, A., Jolson, D., Amundson, K., Buckley, T., Lohse, D., et al. (2012). Design and selection of Toca 511 for clinical use: modified retroviral replicating vector with improved stability and gene expression. Mol. Ther. *20*, 1689–1698. https://doi.org/10.1038/mt.2012.83.

37. Burton, C., Das, A., McDonald, D., Vandergrift, W.A., Patel, S.J., Cachia, D., and Bartee, E. (2018). Oncolytic myxoma virus synergizes with standard of care for treatment of glioblastoma multiforme. Oncolytic Virother. *7*, 107–116. https://doi.org/10.2147/ov.S179335.

38. Zemp, F.J., Lun, X., McKenzie, B.A., Zhou, H., Maxwell, L., Sun, B., Kelly, J.J.P., Stechishin, O., Luchman, A., Weiss, S., et al. (2013). Treating brain tumor-initiating cells using a combination of myxoma virus and rapamycin. Neuro Oncol. *15*, 904–920. https://doi.org/10.1093/neuonc/not035.

39. Wollmann, G., Rogulin, V., Simon, I., Rose, J.K., and van den Pol, A.N. (2010). Some attenuated variants of vesicular stomatitis virus show enhanced oncolytic activity against human glioblastoma cells relative to normal brain cells. J. Virol. *84*, 1563–1573. https://doi.org/10.1128/jvi.02040-09.

40. Chen, Q., Wu, J., Ye, Q., Ma, F., Zhu, Q., Wu, Y., Shan, C., Xie, X., Li, D., Zhan, X., et al. (2018). Treatment of Human Glioblastoma with a Live Attenuated Zika Virus Vaccine Candidate. mBio *9*, e01683-18. https://doi.org/10.1128/mBio.01683-18.

41. Kaid, C., Goulart, E., Caires-Júnior, L.C., Araujo, B.H.S., Soares-Schanoski, A., Bueno, H.M.S., Telles-Silva, K.A., Astray, R.M., Assoni, A.F., Júnior, A.F.R., et al. (2018). Zika Virus Selectively Kills Aggressive Human Embryonal CNS Tumor Cells In Vitro and In Vivo. Cancer Res. *78*, 3363–3374. https://doi.org/10.1158/0008-5472.Can-17-3201.

42. Lubin, J.A., Zhang, R.R., and Kuo, J.S. (2018). Zika Virus has Oncolytic Activity Against Glioblastoma Stem Cells. Neurosurgery *82*, E113–E114. https://doi.org/10.1093/neuros/nyy047.

43. Trus, I., Berube, N., Jiang, P., Rak, J., Gerdts, V., and Karniychuk, U. (2020). Zika Virus with Increased CpG Dinucleotide Frequencies Shows Oncolytic Activity in Glioblastoma. Viruses *12*, 579. https://doi.org/10.3390/v12050579.

44. Zhu, Z., Gorman, M.J., McKenzie, L.D., Chai, J.N., Hubert, C.G., Prager, B.C., Fernandez, E., Richner, J.M., Zhang, R., Shan, C., et al. (2017). Zika virus has oncolytic activity against glioblastoma stem cells. J. Exp. Med. *214*, 2843–2857. https://doi.org/10.1084/jem.20171093.

45. Zhu, Z., Mesci, P., Bernatchez, J.A., Gimple, R.C., Wang, X., Schafer, S.T., Wettersten, H.I., Beck, S., Clark, A.E., Wu, Q., et al. (2020). Zika Virus Targets Glioblastoma Stem Cells through a SOX2-Integrin α(v)β(5) Axis. Cell Stem Cell *26*, 187–204.e10. https://doi.org/10.1016/j.stem.2019.11.016.

46. Pierson, T.C., and Diamond, M.S. (2018). The emergence of Zika virus and its new clinical syndromes. Nature *560*, 573–581. https://doi.org/10.1038/s41586-018-0446-y.

47. Nair, S., Mazzoccoli, L., Jash, A., Govero, J., Bais, S.S., Hu, T., Fontes-Garfias, C.R., Shan, C., Okada, H., Shresta, S., et al. (2021). Zika virus oncolytic activity requires CD8+ T cells and is boosted by immune checkpoint blockade. JCI Insight *6*, e144619. https://doi.org/10.1172/jci.insight.144619.

48. Manokaran, G., Finol, E., Wang, C., Gunaratne, J., Bahl, J., Ong, E.Z., Tan, H.C., Sessions, O.M., Ward, A.M., Gubler, D.J., et al. (2015). Dengue subgenomic RNA binds TRIM25 to inhibit interferon expression for epidemiological fitness. Science *350*, 217–221. https://doi.org/10.1126/science.aab3369.

49. Muruato, A.E., Shan, C., Fontes-Garfias, C.R., Liu, Y., Cao, Z., Gao, Q., Weaver, S.C., and Shi, P.Y. (2019). Genetic stability of live-attenuated Zika vaccine candidates. Antiviral Res. *171*, 104596. https://doi.org/10.1016/j.antiviral.2019.104596.

50. Shan, C., Muruato, A.E., Jagger, B.W., Richner, J., Nunes, B.T.D., Medeiros, D.B.A., Xie, X., Nunes, J.G.C., Morabito, K.M., Kong, W.P., et al. (2017). A single-dose live-attenuated vaccine prevents Zika virus pregnancy transmission and testis damage. Nat. Commun. *8*, 676. https://doi.org/10.1038/s41467-017-00737-8.

51. Li, X.F., Dong, H.L., Wang, H.J., Huang, X.Y., Qiu, Y.F., Ji, X., Ye, Q., Li, C., Liu, Y., Deng, Y.Q., et al. (2018). Development of a chimeric Zika vaccine

CellPress
OPEN ACCESS

using a licensed live-attenuated flavivirus vaccine as backbone. Nat. Commun. *9*, 673. https://doi.org/10.1038/s41467-018-02975-w.

52. Li, X.F., Dong, H.L., Huang, X.Y., Qiu, Y.F., Wang, H.J., Deng, Y.Q., Zhang, N.N., Ye, Q., Zhao, H., Liu, Z.Y., et al. (2016). Characterization of a 2016 Clinical Isolate of Zika Virus in Non-human Primates. EBioMedicine *12*, 170–177. https://doi.org/10.1016/j.ebiom.2016.09.022.

53. Hirsch, A.J., Smith, J.L., Haese, N.N., Broeckel, R.M., Parkins, C.J., Kreklywich, C., DeFilippis, V.R., Denton, M., Smith, P.P., Messer, W.B., et al. (2017). Zika Virus infection of rhesus macaques leads to viral persistence in multiple tissues. PLoS Pathog. *13*, e1006219. https://doi.org/10.1371/journal.ppat.1006219.

54. Todo, T., Ino, Y., Ohtsu, H., Shibahara, J., and Tanaka, M. (2022). A phase I/II study of triple-mutated oncolytic herpes virus G47Δ in patients with progressive glioblastoma. Nat. Commun. *13*, 4119. https://doi.org/10.1038/s41467-022-31262-y.

55. Ferrucci, P.F., Pala, L., Conforti, F., and Cocorocchio, E. (2021). Talimogene Laherparepvec (T-VEC): An Intralesional Cancer Immunotherapy for Advanced Melanoma. Cancers (Basel) *13*, 1383. https://doi.org/10.3390/cancers13061383.

56. Roth, J.C., Cassady, K.A., Cody, J.J., Parker, J.N., Price, K.H., Coleman, J.M., Peggins, J.O., Noker, P.E., Powers, N.W., Grimes, S.D., et al. (2014). Evaluation of the safety and biodistribution of M032, an attenuated herpes simplex virus type 1 expressing hIL-12, after intracerebral administration to aotus nonhuman primates. Hum. Gene Ther. Clin. Dev. *25*, 16–27. https://doi.org/10.1089/humc.2013.201.

57. Hunter, W.D., Martuza, R.L., Feigenbaum, F., Todo, T., Mineta, T., Yazaki, T., Toda, M., Newsome, J.T., Platenberg, R.C., Manz, H.J., and Rabkin, S.D. (1999). Attenuated, replication-competent herpes simplex virus type 1 mutant G207: safety evaluation of intracerebral injection in nonhuman primates. J. Virol. *73*, 6319–6326. https://doi.org/10.1128/jvi.73.8.6319-6326.1999.

58. Kiyokawa, J., and Wakimoto, H. (2019). Preclinical And Clinical Development Of Oncolytic Adenovirus For The Treatment Of Malignant Glioma. Oncolytic Virother. *8*, 27–37. https://doi.org/10.2147/ov.S196403.

59. Bankhead, P., Loughrey, M.B., Fernández, J.A., Dombrowski, Y., McArt, D.G., Dunne, P.D., McQuaid, S., Gray, R.T., Murray, L.J., Coleman, H.G., et al. (2017). QuPath: Open source software for digital pathology image analysis. Sci. Rep. *7*, 16878. https://doi.org/10.1038/s41598-017-17204-5.

STAR★METHODS

KEY RESOURCES TABLE

REAGENT or RESOURCE	SOURCE	IDENTIFIER
Antibodies		
Flavivirus group antigen Antibody (D1-4G2-4-15 (4G2))	Novus Biologicals	cat # NBP2-52709 RRID:AB_2940754
Alexa 488 conjugated Anti-mouse IgG	Thermo-Fisher	cat #A-11029, RRID:AB_2534088
CD3 [LN10]	Biocare Medical	API3152AA
Human CCR2 Antibody	R&D Systems	cat #MAB150-100, RRID:AB_2247178
CD11c Antibody (BU15)	Novus Biologicals	cat #NBP1-45018, NBP1-45018
Anti-CD8 alpha antibody [EPR21769] (ab217344)	Abcam	cat #ab217344, RRID:AB_2890649
HRP conjugated anti-monkey IgG	Sigma	cat #A2054, RRID:AB_257967
Bond Intense R Detection System	Leica	DS9263
Iba-1 antibody	Fujifilm	cat #019–19741, RRID:AB_839504
Bacterial and virus strains		
Δ10 3′-UTR ZIKV	Laboratory of Pei-Yong Shi	NA
Biological samples		
Brain tissue	C57BL/6 mice, see experimental model and subject details below.	NA
Blood, Urine from day −2, 2, 3, 6,7,10 and 14 after intracranial injection	Rhesus macaques	NA
Postmortem: brain, sciatic nerve, brachial plexus, trigeminal ganglion, spinal cord (cervical, thoracic, and lumbar), eye, kidneys, bladder, uterus, vagina, cervix, knee, elbows, lungs, spleens, cervical, inguinal, mesenteric, axillary lymph nodes, bone marrow, colon, ileum, duodenum, jejunum, stomach and liver.	Rhesus macaques	NA
Chemicals, peptides, and recombinant proteins		
RNA later	Thermo-Fisher	AM7021
TRIzol reagent	Thermo-Fisher	15596–018
TaqMan One-step RT-PCR reagent	Thermo-Fisher	11732927
o-phenylenediamine dihydrochloride (ELISA substrate)	Thermo-Fisher	11732927
Critical commercial assays		
Rnascope® 2.5 HD Reagent Kit - BROWN	ACD	322300
DMEM	Thermo Fisher	MT-10-013-CV
Fetal bovine serum	VWR	89510–188
Experimental models: cell lines		
C6/36 cells	ATCC	CRL-1660, RRID:CVCL_Z230, RRID:CVCL_Z230
Vero cells	ATCC	CCL-81, RRID:CVCL_0059, RRID:CVCL_0059
SB28 cells	Laboratory of Hideho Okada, UCSF	NA, RRID:CVCL_A5ED
CT-2A cells	Millipore Sigma	SCC194, RRID:CVCL_ZJ44
Experimental models: organisms/strains		
Rhesus macaques	Oregon National Primate Research Center (ONPRC)	NA
C57Bl/6 mice	Jackson laboratories	000664, RRID:IMSR_JAX:000664
Software and algorithms		
QuPath	Bankhead et al.[59]	NA

EXPERIMENTAL MODEL AND SUBJECT DETAILS

Ethics statement regarding non-human primate research

All Δ10 3′-UTR ZIKV infection experiments using animals were performed in compliance with guidelines established by the Animal Welfare Act for housing and care of laboratory animals and conducted in accordance with Oregon National Primate Research Center (ONPRC) Institutional Animal Care and Use Committee approved protocol (IACUC #3459). Rhesus macaque studies were performed in ABSL-2 containment facilities at the Oregon National Primate Research Center (ONPRC), which are accredited by the Assessment and Accreditation of Laboratory Animal Care (AAALAC) International. Appropriate procedures were utilized to reduce potential distress, pain and discomfort. Ketamine (10 mg/kg) was used to sedate the animals during all procedures including routine blood draws performed by trained veterinary staff. Rhesus monkeys were fed standard monkey chow twice daily and the amount was matched to each animal according to body weight, age and sex and intake was monitored. Animals also received daily food supplements and other enrichment devices. The infected animals were caged with partners or separately but within visual and auditory contact of other animals to promote social behavior. The animals were monitored postoperatively for at least 7 days by a member of the Surgical Services Unit. Their appetite, stool, attitude, incision site and any indication of pain and discomfort were monitored daily. This monitoring was documented daily in each animal's record. The animals had a decreased appetite postoperatively which is common with opioid analgesia, during that time the animals are offered additional fruits, vegetables, and enrichment. This postoperative monitoring is in addition to the routine daily monitoring by Operations staff of all animals in the facility for any clinical or behavioral abnormalities. These six animals were noted for nasal discharge prior to necropsy. Cultures and sensitivities were obtained in case clinical treatment was indicated. None of the animals required clinical treatment. The animals were monitored by the Clinical Medicine Staff during the time of nasal discharge. Several of the culture results were positive for Moraxella. There were other animals in the same facility with similar clinical signs. Moraxella is a mild upper respiratory infection that we see annually in the colony usually at this time of year. The nasal discharge from the animals did not appear study related and did not require treatment. The clinical monitoring is documented daily in the animals' record. Post operatively and clinically there were no significant differences noted between the six animals. At the designated time points, the animals were euthanized according to the recommendations of the American Veterinary Medical Association 2013 panel on Euthanasia.

Virus

ZIKV strain FSS13025 containing a previously described 10 nucleotide deletion in the 3′ untranslated region[47] was propagated on C6/36 cells.[49] Supernatant was collected at 10 dpi, centrifuged at 1,000 x g for 10 min, and filtered through a 0.45 μm filter to remove cellular debris. Virus was then concentrated and purified by ultra-centrifugation through a cushion of 20% sorbitol (50 mM Tris 1mM $MgCl_2$ pH 8.0) at 150,000 x g for 2 h at 4°C. Pellets were resuspended in 1/100th of the original volume in PBS and stored at −80°C until use.

Mouse brains

8- to 9-week-old mice were anesthetized and intracranially injected with a single cell suspension of SB28 or CT2A cells using a stereotactic apparatus (Stoelting). At day 7 after tumor implantation, mice with similar flux were randomized between groups. Using the same coordinates as for tumor implantation, mice were inoculated intratumorally with mouse-adapted ZIKV (10^5 FFU).[47] At 21 days after tumor implantation, we perfused mice with 4% paraformaldehyde (PFA) and collected their brains. The brains were then embedded in paraffin and sectioned into 5 μm-thick coronal sections.

Non-human primates

All six *Macaca mulatta* were females, aged 7–11 years. Prior to studies, they were tested and confirmed negative for prior anti-ZIKV antibodies by ELISA.

METHOD DETAILS

Magnetic resonance imaging (MRI)-guided stereotactic surgery

Pre-operative care consisted of overnight food restriction (standard operating procedure prior to surgery) for 12 h and general examination to ensure that primate health is adequate to withstand the procedure. On the day of stereotaxic surgery, animals were anesthetized with ketamine HCl, followed by maintenance anesthesia with isoflurane gas vaporized in 100% oxygen. An MRI was conducted to obtain proper surgical coordinates (Figure S1). Animals were taken directly from MRI to the operating room. For stereotaxic surgery and virus injection, the cranial bone was exposed, and the anterior/posterior location determined by positioning the instrument (injection needle) at ear bar zero. One hole (approximately 0.5 cm) over the right frontal lobe was made using an air drill to expose the dura. A Hamilton syringe fitted with a 25-gauge needle, mounted on the micromanipulator of the stereotaxic instrument,

was used to inject 5×10^6 FFU of Δ10 3′-UTR ZIKV in 100 μL of PBS or 100 μL of PBS only. The virus was injected into the right frontal lobe of each animal. The rate of injection was maintained at 2–3 μL/min using an infusion pump. The needle was left in place for an additional 5 min to allow the injectate to diffuse from the needle tip. After the microinjection was completed, the skull opening was filled with gelfoam and the incision closed.

Preparation of NHP brain sections

At necropsy, the brain was perfused with PBS prior to removal. Samples from other major organs and lymph nodes, were collected and stored in RNAlater (Invitrogen) or TRIzol (Invitrogen) for RNA isolation. Each brain was cut coronally into 4 mm slabs. 2–3 pairs of 2 mm diameter punches, equally divided between hemispheres, were collected for detection of viral RNA and infectious virus (exemplified in Figures 2A, S2, and S3). The slabs were then transferred to 4% PFA for 48 h, moved to an overnight wash in PBS, placed in 15% sucrose until the tissue sunk, then 30% sucrose until tissue sunk. The blocks were then mounted on a freezing microtome stage atop a flat sheet of OCT. Once the entire block was frozen, they were sectioned to 20 μm and stored at −80°C in a cryoprotectant solution.

QUANTIFICATION AND STATISTICAL ANALYSIS

Immunohistochemistry

Every tenth NHP section of block 4 of each of the animals (block where injection was performed) was thawed and mounted on gelatin-coated slides. The slides were air-dried then vacuum-dried overnight to increase section adhesion. The first group of serially mounted slides was used for hematoxylin and eosin staining. Using the blocks where signs of inflammation were detected, a second group of one in every tenth sections was serially mounted and immunostained with rabbit anti-IBA1 (1:500, Wako Chemicals) prior to species-specific secondary antibody incubation and development using Vectastain Elite ABC kit (Vector Laboratories). A third group of serially mounted brain sections was used for CD3 staining (1:200, Novus Biologicals). A fourth group of serially mounted brain sections was stained using a cross-reactive anti-flavivirus E protein antibody (1:200, Novus Biologicals) for detection of ZIKV E protein in the brain tissue. A fifth group of sections was stained using an anti-CCR2 antibody (1:100, R&D Systems) and a sixth group using an anti-CD11c antibody (1:200, R&D Systems).

One section from block 5, containing subventricular region was stained using RNAScope with the previously mentioned anti-ZIKV probe to assess the presence of viral RNA in a region rich in neuronal stem cells. The hematoxylin and eosin and RNAscope stainings were repeated with the central most 20 μm section of blocks containing the subventricular zone, and hippocampus.

The murine brain sections from SB28 tumors were stained using the anti-flavivirus E protein antibody (1:200, Novus Biologicals), and the sections from mice injected with ZIKV after either CT2A cells or PBS injections were stained using an anti-CD8 antibody (1:1000, abcam).

All brain sections were then scanned on a NanoZoomer 2.0-HT slide scanner (Hamamatsu Photonics K.K.) with a 20x objective. The percent of IBA1-positive area and CD3, CCR2, CD11c and CD8-expressing cells was determined using QuPath software.[59]

Detection of infectious virus in brain

Tissues were homogenized in 0.5 mL of DMEM cell culture medium containing 5% FBS and PSG plus approximately 250 μL of SiLiBeads using a bead beater (Precellys 24 homogenizer, Bertin Technologies), and cellular debris were pelleted by centrifugation (5,000 × g for 2 min). A 100 μL sample of the clarified lysate was applied to a T-25 flask of C6/36 cells for seven days. Supernatant titers from these cultures were transferred to Vero cells, incubated at 37°C for an additional 3 days and assayed for the presence of infectious virus by focus assay using mAb anti-ZIKV E (envelope) protein and an anti-mouse IgG horseradish peroxidase-conjugated secondary antibody.

Quantification of viral RNA

RNA from brain punches, tissue samples, blood, urine, and cerebrospinal fluid (CSF) was isolated using TRIzol (Invitrogen) according to the manufacturer's protocol. ZIKV RNA levels were measured by a one-step quantitative real time reverse transcription polymerase chain reaction assay (RT-qPCR) using TaqMan One-Step RT-PCR Master Mix (Thermo Fisher Scientific), as previously described.[53]

Viral RNA *in situ* hybridization

A group of 20 μm thick serially mounted sections from the blocks where ZIKV RNA was detected by qRT-PCR was stained using the 2.5 HD Assay - BROWN kit according to manufacturer's protocol (ACDBio). Briefly, after incubation with hydrogen peroxide, the brain sections underwent target retrieval for 15 min followed by washes and incubation with protease plus and a ZIKV RNA specific probe (catalog #467771). Following amplification of the signal, the brains were incubated with a 1:1 mix of Brown A and Brown B solutions, followed by 50% hematoxylin and, finally, mounted using Cytoseal (Espredia).

ELISA

Enzyme-linked immunosorbent assays were performed as previously described.[53] Briefly, high-binding 96-well plates (Corning) were coated with 10^6 FFU per well of purified ZIKV (PRVABC59) diluted in PBS. (*i.e.*, not the same virus used for inoculation), blocked with 2% milk/PBS +0.05% Tween, then incubated with 3-fold dilutions of plasma starting at a dilution of 1:50. HRP-conjugated secondary antibodies (anti-monkey total IgG) were used at 1:1000 concentration. Bound secondary antibody was detected using the OPD substrate (Life Technologies) followed by HCl to stop the assay. The plates were read within 10 min using a Synergy HTX Microplate Reader (BioTek) at 490 nm. Endpoint titers of ZIKV binding antibodies were determined using a Log/Log transformation method.

Statistical analysis

All data were analyzed using GraphPad Prism 8 software. Data between two groups were analyzed by unpaired two-tailed Student's *t*-tests. Statistical significance was set at $p < 0.05$.

Cell Reports Medicine, Volume *6*

Supplemental information

Intracranial injection of genetically modified, mosquito non-transmissible Zika virus: Safety in primates and ramifications for brain tumor therapy

Alec J. Hirsch, Amanda de Andrade Costa, Cody German, Christopher J. Parkins, Jessica L. Smith, Emilie Russler-Germain, Ashwani Kesarwani, Yuping Li, Verginia Cuzon Carlson, Timothy Carlson, Jodi L. McBride, Sathya Srinivasan, Anne D. Lewis, Xuping Xie, Pei-Yong Shi, Michael S. Diamond, and Milan G. Chheda

Supplementary Figures

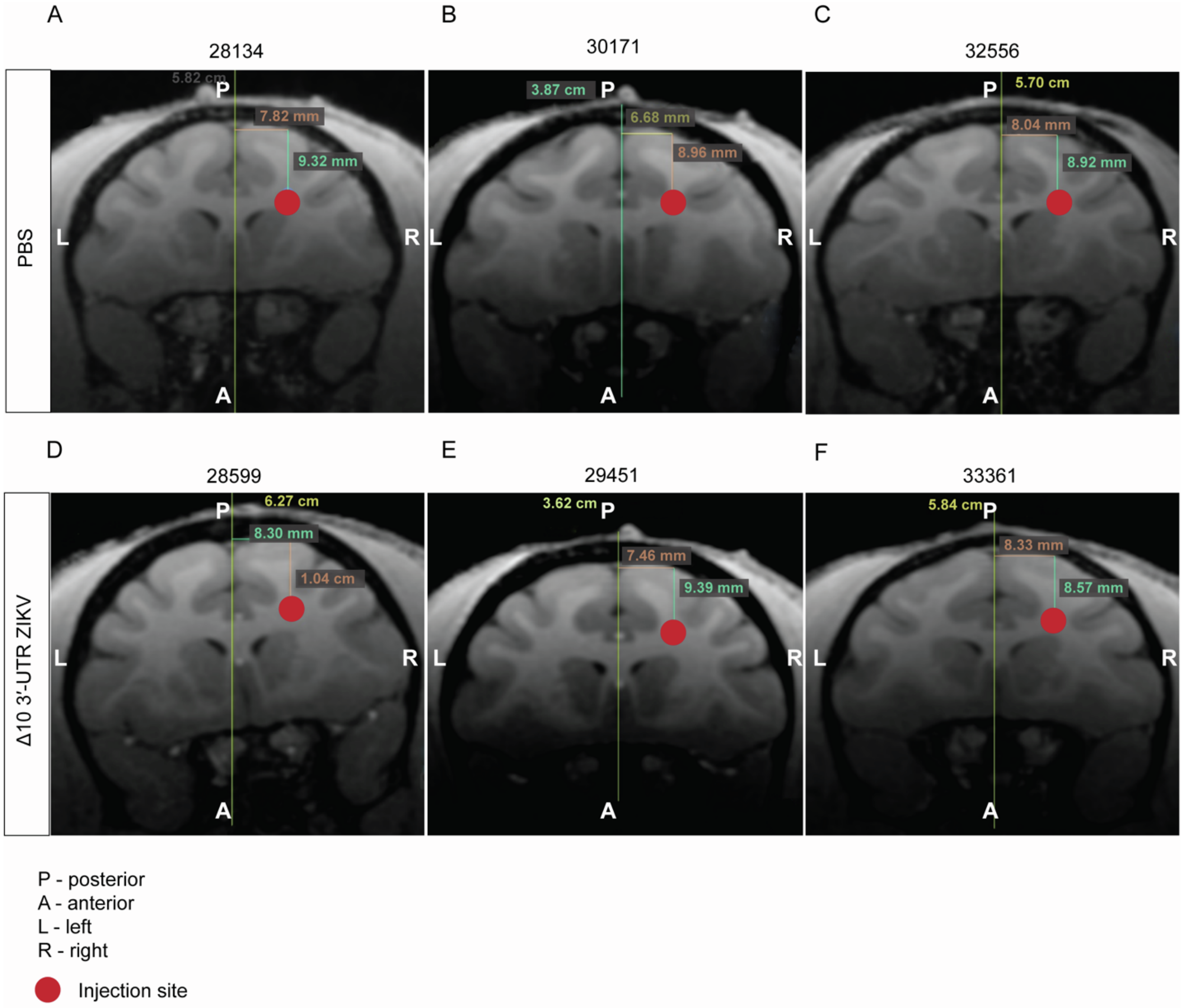

Figure S1. MRI-guided injection coordinates. MRI scan indicating the exact coordination for injection of PBS (A, B, C) or Δ10 3′-UTR ZIKV (D, E, F) for animals A. 28134 B. 30171 C. 32556 D. 28599 E. 29451 F. 33361. Related to Figure 1.

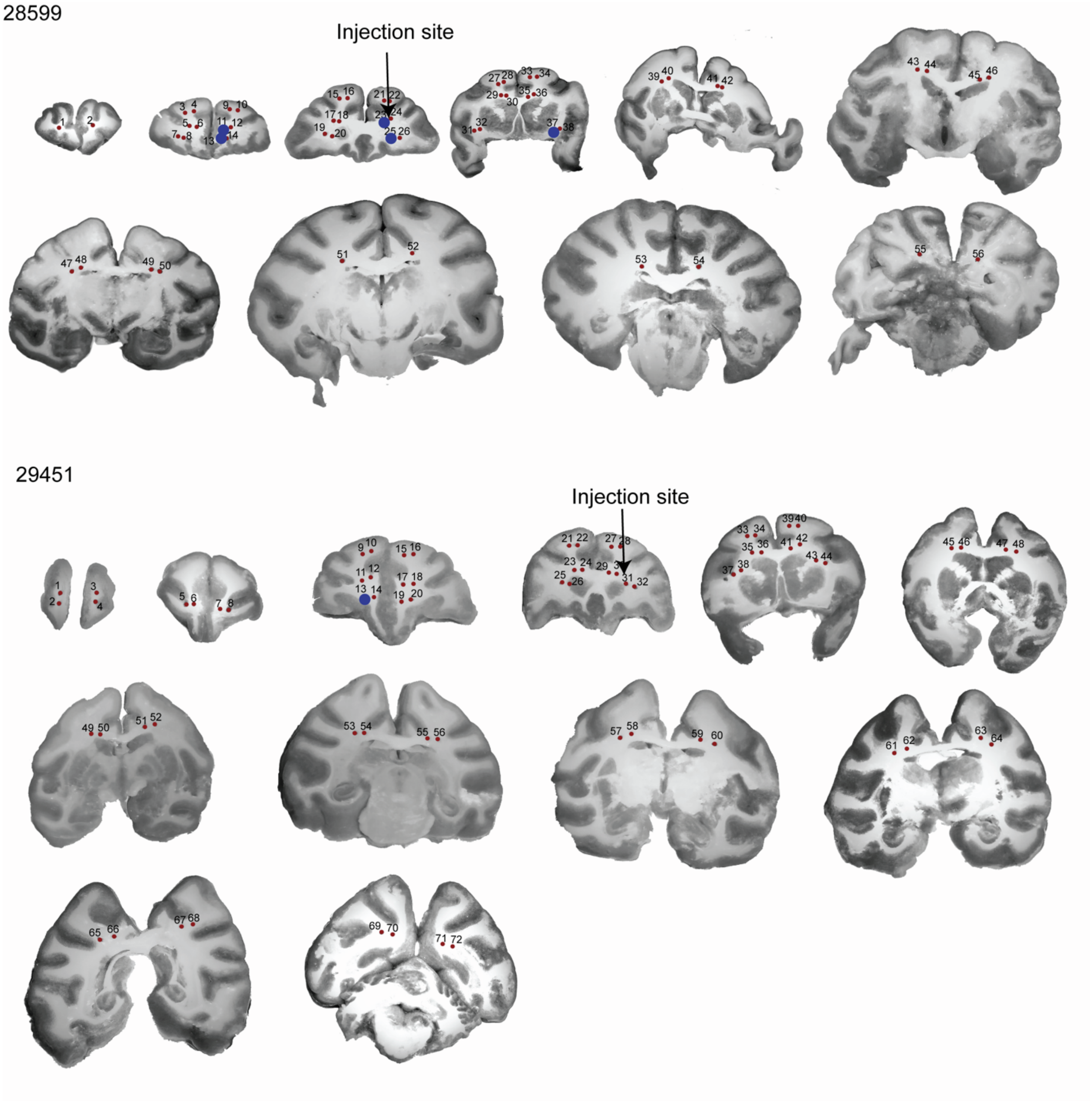

Figure S2. Representation of brain slabs from ZIKV injected macaques with numbered sampling sites indicated. Related to Fig 2.A.

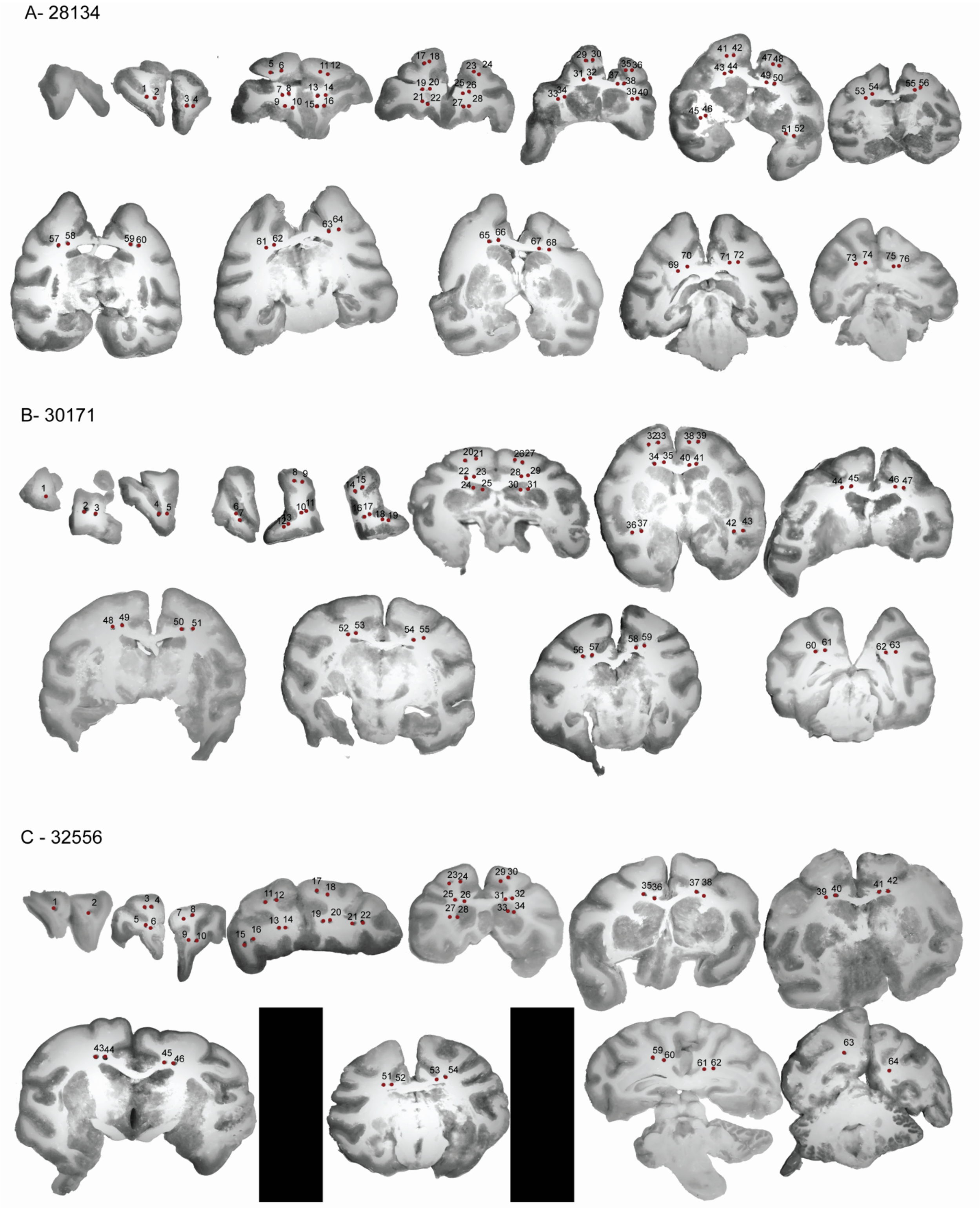

Figure S3. Representation of brain slabs from PBS injected macaques with numbered sampling sites indicated. Related to Figure 2A.

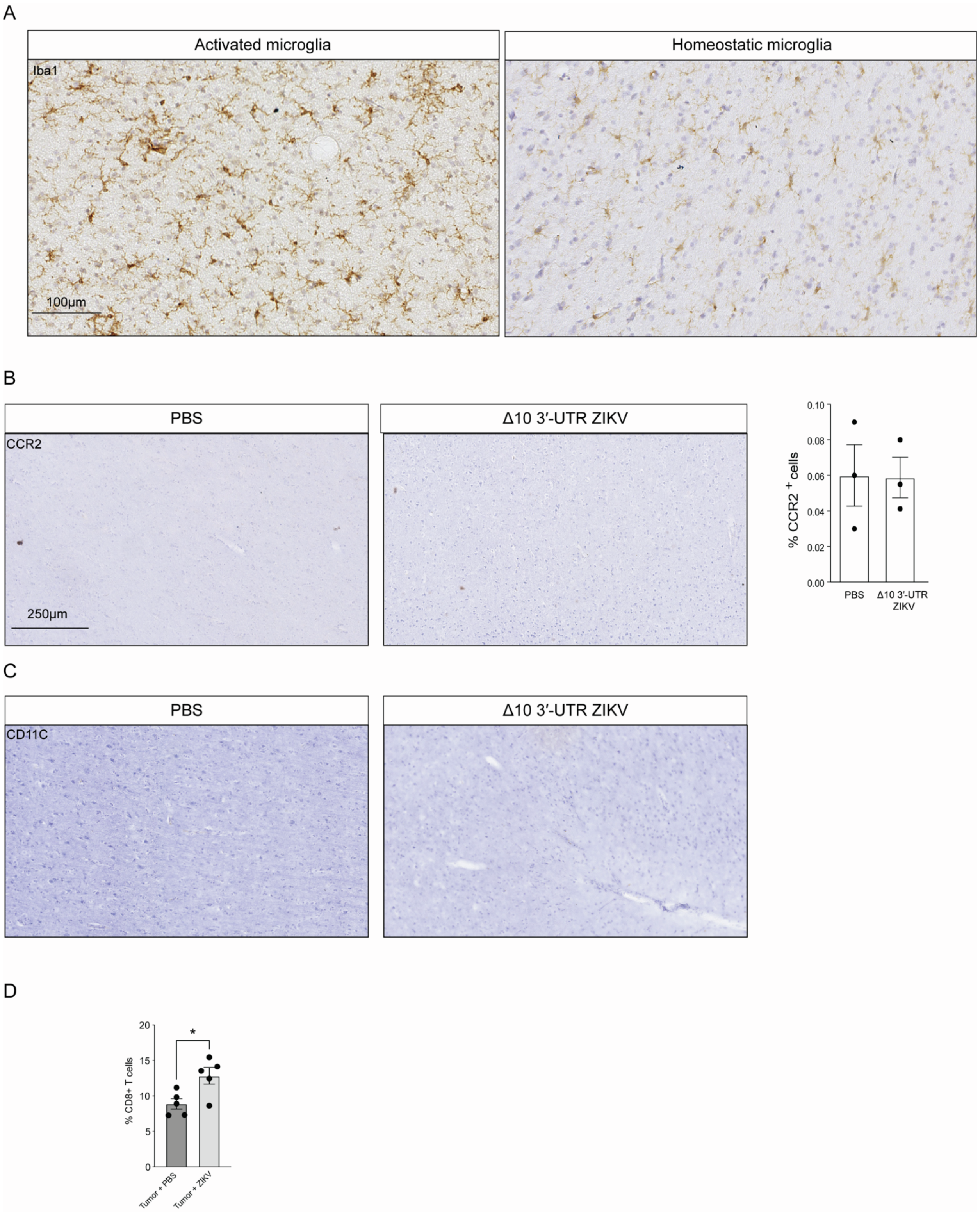

Figure S4. Immune cell populations in the brain after intracerebral ZIKV injection.

A. IBA1 immunostaining of Δ10 3′-UTR ZIKV injected macaques demonstrated focal areas where microglia showing an activated phenotype (left panel) versus their basal

morphology (right panel). **B.** Quantification of CCR2-expressing cells in the brains of NHP after intracranial injection of PBS or ZIKV. **C.** Quantification of CD11c-expressing cells in the brains of rhesus macaques after intracranial injection of PBS or ZIKV. **D.** Quantification of expression of CD8-expressing cells in the brains of na.ve mice, and brains with or without tumors 14 days after ZIKV injection. Related to Figure 3.
